# Transitions in and out of Loneliness During the COVID‐19 Pandemic: A Latent Class Analysis of Older Adults in England

**DOI:** 10.1111/1467-9566.70138

**Published:** 2025-12-21

**Authors:** Mengxing Joshi, Daniela Weber, Anne Goujon

**Affiliations:** ^1^ School of Geography and Sustainable Development University of St Andrews St Andrews UK; ^2^ International Institute for Applied Systems Analysis Vienna Austria; ^3^ Vienna University of Economics and Business Vienna Austria

**Keywords:** English Longitudinal Study of Ageing, inequality, pandemic loneliness, population ageing, social health

## Abstract

Disease control measures during the COVID‐19 pandemic may have intensified loneliness among older adults, though experiences varied based on individual vulnerabilities and resources. This study examines loneliness trajectories among older adults using the English Longitudinal Study of Ageing, spanning four waves: two pre‐pandemic (Wave 8: 2016–2017; Wave 9: 2018–2019) and two COVID‐19 substudies (June–July and November–December 2020). The sample included 4492 respondents (17,968 observations). Latent class growth analysis identified four loneliness trajectories: ‘not lonely’ (73.5%), ‘pandemic loneliness’ (12.7%), ‘transitioned out of loneliness’ (6.9%) and ‘enduring loneliness’ (6.8%). Multinomial regression analysis explored predictors of trajectory membership. Younger age (50–74), being female, depression, COVID‐related worries and disrupted daily routines increased the likelihood of belonging to ‘pandemic loneliness’ rather than ‘not lonely’. Optimism and strong partner support increased the likelihood of remaining ‘not lonely’ or transitioning out of loneliness. The pandemic's unintended effects, including routine disruptions and financial concerns, heightened loneliness risks, whereas psychosocial resources provided critical resilience. To prepare for future public health crises, policies should strengthen mental health support, promote social and economic stability and enhance social connection and resilience. Addressing psychosocial factors is essential to reducing loneliness and protecting older adults' well‐being during and beyond periods of crisis.

## 1 Introduction

Loneliness is an unpleasant and distressing emotion resulting from deficiencies in social relationships that may arise when disparities exist between the social connections people would like to have and those they actually have (Peplau and Perlman 1982). Research has shown that loneliness is associated with physical health conditions (e.g., high blood pressure, coronary heart disease, Type 2 diabetes and reduced immune functioning), poorer mental health and cognitive function, increased morbidity and mortality and diminished quality of life (Park et al. 2020). In the EU Loneliness Survey 2022, more than one third of respondents reported being lonely at least sometime (Berlingieri et al. 2023).

The COVID‐19 pandemic has brought loneliness more prominently into the public discourse. Disease control measures, such as quarantine, lockdowns, shielding policies and social distancing, have significantly disrupted people's social lives, potentially resulting in increased social isolation, which refers to the objective lack of social contacts (Valtorta and Hanratty 2012), and heightened levels of loneliness (Buffel et al. 2023). Older adults are often considered vulnerable to loneliness due to ageing‐related risk factors, such as widowhood, mobility limitations, health deterioration and cognitive impairment (Kemperman et al. 2019). They also faced a disproportionate impact from shielding policies and stay‐at‐home advice due to their higher vulnerability to the virus. Considering these dual challenges, it is crucial to determine whether there has been a significant increase in loneliness among the older population during this period.

Existing longitudinal evidence yields mixed results, which is unsurprising given the considerable differences in COVID‐19 restriction measures implemented by different nations (Stolz et al. 2021). For instance, studies in the Netherlands (van Tilburg et al. 2021) and Austria (Stolz et al. 2021), where strict lockdown measures were enforced, reported an increase in loneliness among older adults during the pandemic. Studies in the United States (Peng and Roth 2022), which implemented varying levels of stay‐at‐home orders across states, and Sweden (Kivi et al. 2021), which remained open with more voluntary measures, found that levels of loneliness remained stable among older adults. In the UK context, several studies examined changes in loneliness among older adults before and during the pandemic using data from the English Longitudinal Study of Ageing (ELSA). Zaninotto et al. (2022) and Chatzi and Nazroo (2021) reported increases in loneliness. Mansfield et al. (2024) found a slight increase in the percentage of older adults reporting loneliness without isolation, as well as those experiencing both loneliness and social isolation. Kung and Steptoe (2024) compared changes in loneliness between older adults who were not isolated before the pandemic and those who were previously isolated, finding a greater increase in loneliness among the former group. However, these studies treat loneliness as a single average effect and do not account for heterogeneity within the older population. Not all older adults have weathered the pandemic with the same set of advantages and resources. They have undergone distinct experiences throughout the pandemic, encompassing disparities in health issues, age‐related discrimination, financial exclusion and pressures on mental well‐being (Buffel et al. 2023). The loneliness experiences and outcomes may also vary significantly among them. Therefore, understanding heterogeneity in older adults' loneliness experiences is crucial to avoid either underestimating or overestimating the negative impacts of the pandemic on their social well‐being.

A limited number of studies have examined different loneliness trajectories during the pandemic. Mayerl et al. (2022) identified four mental health trajectories among older adults in Austria across three pandemic phases (i.e., May 2020, March 2021 and December 2021): a resilient class with low loneliness, depressive and anxiety symptoms; an increasing burden class with rising depressive and anxiety symptoms and stable high loneliness; a recovered class with decreasing depressive and anxiety symptoms and stable low to mediocre loneliness; and a high burden class with no considerable changes but generally high levels across all three mental health constructs. In the UK context, Bu et al. (2020) reported four loneliness trajectories among all‐age adults during the strict lockdown (i.e., March 23 to May 10, 2020): one class with the highest baseline loneliness and increased loneliness, one class with the lowest baseline loneliness and decreased loneliness and another two classes with middle baseline loneliness and relatively constant loneliness. However, this study does not specifically focus on older people, lacks a pre‐pandemic baseline measure of loneliness and does not incorporate pandemic‐related factors, such as self‐isolation at home, experiences of COVID‐19 symptoms or worries triggered by the pandemic when predicting trajectory membership.

An additional limitation within the existing literature on the pandemic's impact on loneliness is the lack of comprehensive studies that simultaneously consider three key dimensions: pre‐pandemic general risk factors associated with loneliness (Dahlberg et al. 2022), pandemic‐related factors that may exacerbate loneliness (Stolz et al. 2021) and protective factors that could potentially help older adults mitigate the impact of the pandemic (Fuller and Huseth‐Zosel 2021). As suggested by a recent meta‐analysis of longitudinal studies on the pandemic's impact on loneliness, there is a pressing need for further investigation into risk and protective factors to explain the heterogeneity of pandemic effects on loneliness to inform the development of targeted intervention (Ernst et al. 2022).

In summary, to our knowledge, there has been no study thus far that comprehensively examines the heterogeneous trajectories of loneliness changes during the pandemic in comparison to pre‐pandemic baselines, specifically within the older population in the UK context, while simultaneously exploring all three categories of factors—general and pandemic risk factors, as well as protective factors. Our study addresses this notable research gap through answering the following research questions (RQ):

RQ1: What are the heterogeneous trajectories of loneliness experienced by people aged 50 and over in England in the COVID‐19 pandemic compared to before the pandemic?

RQ2: What are the general risk factors, pandemic‐related factors and protective factors associated with these observed loneliness trajectories?

## 2 Three‐Pillar Framework for Distinctive Loneliness Trajectories

**FIGURE 1:**
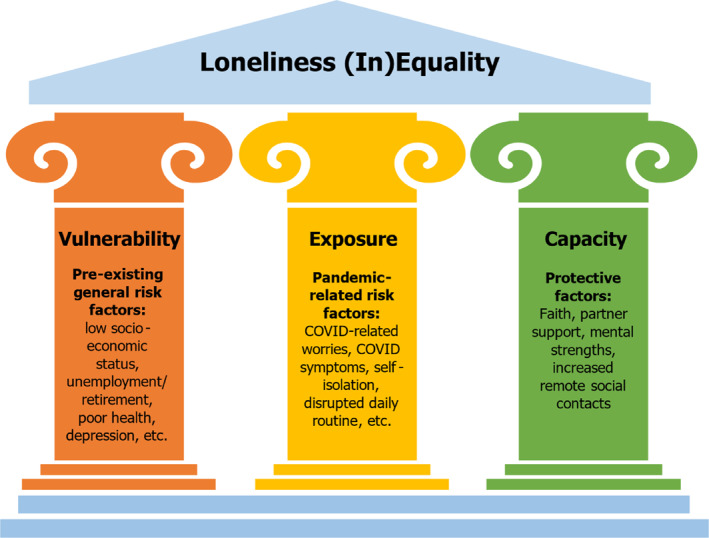
Three‐pillar framework for distinctive loneliness outcomes during the pandemic.

In this study, we hypothesise that distinct patterns of loneliness trajectories exist within the older population in response to the pandemic. To explore this, we introduce a three‐pillar theoretical framework (Figure 1), which encompasses (1) existing vulnerability to loneliness, (2) exposure to pandemic‐related factors and (3) one's capacity to respond by coping and adaptability. This framework is conceptualised by the authors; however, the selection of factors within each pillar is grounded in established empirical evidence on loneliness and resilience, as elaborated below. The first pillar, existing vulnerabilities, highlights the baseline risks faced by older adults prior to the pandemic. Factors such as poor self‐rated health, depression (Dahlberg et al. 2022) or socioeconomic disadvantages such as low income (Donovan et al. 2017) and unemployment (Lasgaard et al. 2016), limit social connections and make older adults more susceptible to loneliness. These vulnerabilities reflect psychosocial inequalities that position certain individuals at greater risk, even before pandemic‐specific stressors come into play. The second pillar, exposure to pandemic‐related factors, examines how the pandemic amplified loneliness risks through its unintended consequences. Experiencing COVID‐19 symptoms and self‐isolating at home can reduce social interactions, increasing the risk of loneliness (Li and Wang 2020). Lockdown measures can also trigger additional stressors, such as worries about inadequate supplies and financial loss. Worries, defined as repetitive thoughts about future events (Borkovec 1994), likely exacerbated loneliness by heightening psychological stress (Anyan et al. 2020). Moreover, lockdown‐induced disruptions to daily routines have been linked to higher levels of depression, reduced quality of life and decreased life satisfaction (Di Gessa and Zaninotto 2023), contributing to loneliness among older adults. The third pillar, capacity to respond, reflects an individual's power and available resources to modify exposures and effectively manage, adapt to and recover from their effects (Diderichsen et al. 2019). Older adults with greater access to resources are expected to better navigate restrictions and stressors during the pandemic, reducing their risk of increased loneliness (Dahlberg 2021). Factors such as optimism about the future, faith and partner support played crucial roles for older adults in enhancing coping and adaptability during this period (Fuller and Huseth‐Zosel 2021). Non‐physical social contacts, such as phone and video calls, also emerged as key protective factors, enabling older adults to maintain social connections despite reduced in‐person interactions (Arpino et al. 2021). The different occupation of these resources can lead to the perpetuation of disparities in loneliness outcomes during the pandemic, with some older adults better positioned to navigate pandemic‐related challenges than others. Together, these three pillars (vulnerability, exposure and capacity) offer a comprehensive framework to understand the role of pre‐existing inequalities, pandemic‐specific stressors and individual resilience in contributing to the heterogeneous trajectories of loneliness among older adults during the pandemic.

## 3 Method

### 3.1 Study Design and Participants

**FIGURE 2:**
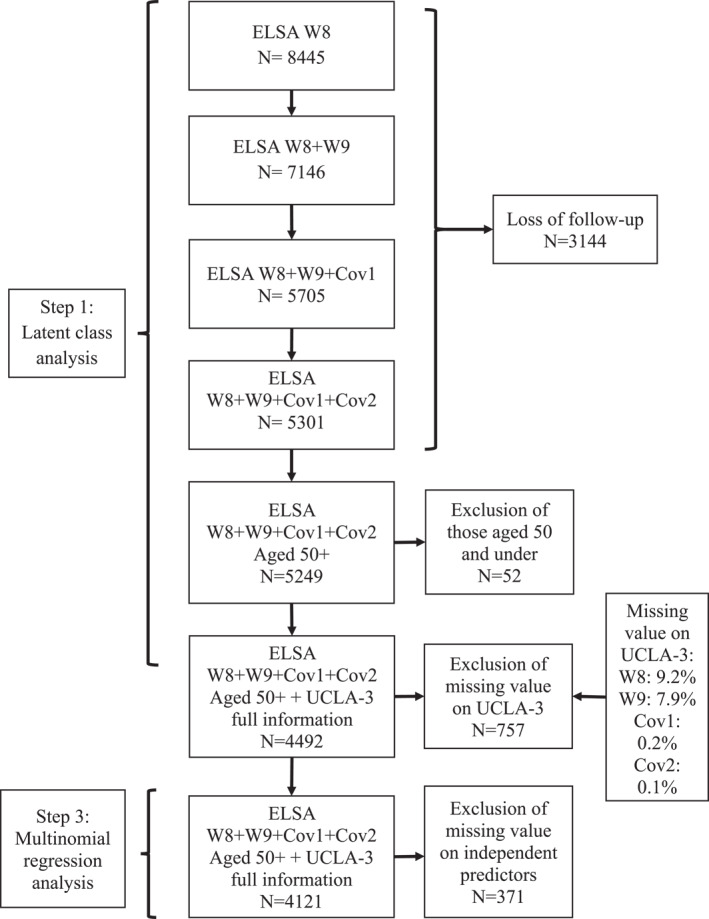
Flow chart of participant inclusion in the study.

This study draws upon data from the English Longitudinal Study of Ageing (ELSA), a nationally representative panel study comprising individuals aged 50 and over living in noninstitutionalised households in England. Further details about ELSA can be found in Banks et al.’s (2021) report. ELSA contains two waves of the COVID‐19 Substudy, conducted via online surveys or telephone interviews at two time points: June–July 2020 and November–December 2020 (Addario and Wood 2022). These periods correspond to distinct phases of the UK pandemic response, with the first following the easing of the initial national lockdown and the second coinciding with a renewed lockdown amid rising infection rates. Further contextual details about national restrictions and case trends are provided in Supporting Information S1: Material 1. Our longitudinal analysis includes data from the two most recent pre‐pandemic waves, Wave 8 (2016–2017) and Wave 9 (2018–2019), and the two waves of the COVID‐19 Substudy, which are referred to as Cov1 and Cov2 for clarity. Our analysis includes all participants aged 50^1^ and over who participated in all four waves and responded to questions related to loneliness, yielding a final sample of 4492 individuals with 17,968 observations. Figure 2 reports the process of participant selection and inclusion in our study.

### 3.2 Measurements

*Loneliness* was measured across all four waves using the three‐item University of California, Los Angeles (UCLA) loneliness scale (Hughes et al. 2004). Respondents were asked how often they felt ‘lack of companionship’, ‘left out’ and ‘isolated’, which were scored as 1 (hardly ever or never), 2 (some of the time) and 3 (often). Their sum yielded a loneliness scale ranging from 3 to 9. There is no universally accepted threshold for which a person would definitely be considered lonely (Office for National Statistics, 2018). In line with previous studies (Steptoe et al. 2013; C. R. Victor and Pikhartova 2020; Pikhartova et al. 2016), the scores were dichotomised, with scores of 3–5 categorised as not lonely and 6–9 classified as lonely. Supporting Information S1: Material 2‐a shows how specific response patterns correspond to dichotomised loneliness scores. Supporting Information S1: Material 2‐b presents the sensitivity analysis using a three‐category classification in latent class analysis.

For demographic factors and general risk factors, we use data from Wave 8 as we aim to capture the baseline risks faced by older adults before the pandemic and identify who is at risk for future events. Pandemic‐related risk factors and protective factors were drawn from Cov1 (wherever available), as the latent class analysis identified a significant change point at this wave. This makes Cov1 the most appropriate wave for capturing the immediate impacts and conditions relevant to the trajectories identified.

#### 3.2.1 Vulnerability Pillar: General Risk Factors

We use *subjective social status (SSS)* as a measure of socioeconomic status, a question asking participants to place themselves on a ladder to represent their social status. Supporting Information S1: Material 3‐a describes the SSS question in detail. The SSS score was divided into tertiles labelled as low, middle and high SSS. *Employment status* was categorised into three groups: ‘retired’, ‘employed’ and ‘not‐in‐labour‐force’ (including unemployed, permanently sick or disabled, or looking after home or family). *Self‐rated general health* was grouped into ‘good’ (excellent, very good and good) and ‘poor’ (fair and poor), consistent with previous studies using ELSA data (Pikhartova et al. 2016; Prakash et al. 2025). We also tested the sensitivity of self‐rated general health with the binary measure and the original five‐category Likert scale. The results did not differ. *Depression* was measured using the eight‐item version of the Center for Epidemiologic Studies Depression Scale (CES‐D‐8). Each item had a dichotomous response format (yes‐1/no‐0), with two positively worded items reverse coded (yes‐0/no‐1), producing a total score ranging from 0 (no symptoms) to 8 (all eight symptoms). A total score of 3 or higher indicates elevated depressive symptoms, consistent with previous ELSA‐based studies on loneliness and mental health (Pikhartova et al. 2016; White et al. 2016; Steptoe et al. 2013). Further details of this measure are provided in Supporting Information S1: Material 3‐b and 3‐c.

#### 3.2.2 Exposure Pillar: Pandemic‐Related Risk Factors

If respondents reported worries regarding any of the following: future financial situation, enough food and other essential items, they were categorised as having *COVID‐related worries*. *Disrupted daily routines* were defined based on participants' reports of two or more of the following items: decreased physical activity, increased/decreased eating and increased/decreased sleeping. This measure captures disturbances in the structure and rhythm of everyday life during the pandemic, which are linked to emotional well‐being and loneliness (Ratcliffe et al. 2022). Research findings suggest that individuals who reported any changes, whether increases or decreases, in their eating and sleeping behaviours were more likely than those who maintained unchanged habits to report higher levels of depression, lower quality of life and reduced life satisfaction during the COVID‐19 pandemic (Di Gessa and Zaninotto 2023). Therefore, we classify any direction of change in eating or sleeping as disrupted daily routines. Experiencing two or more COVID symptoms was coded as having *COVID symptoms*. *Self‐isolation* in April/June was coded as yes or no based on whether individuals reported not leaving home for any reason, including not going out to buy food and not seeing people outside of the household.

#### 3.2.3 Capacity Pillar: Protective Factors

*Pray/meditate daily* was sourced from Wave 9 because it is not available in the COVID Substudy. *Optimism about the future* was measured using the Self‐realisation subscale of the Control, Autonomy, Self‐realisation and Pleasure (CASP‐12) scale in Cov1 (see Supporting Information S1: Material 3‐d). The total score ranges from 0 to 9, with higher scores indicating greater optimism (Cronbach's alpha = 0.82). *Partner's emotional* *support*, measured in Cov1, includes three questions: ‘My partner understands the way I feel about things’; ‘I can rely on partner if I have a serious problem’; and ‘I can open up to my partner if they need to talk about my worries’, with answers ‘a lot’, ‘some’, ‘a little’ and ‘not at all’. This was categorised as ‘high support’ if participants reported ‘a lot’ or ‘some’ for all three questions; otherwise, it was classified as ‘low support’. Participants without a partner were labelled as ‘no partner’. In‐person meetings were not assessed in Cov1 but were included in Wave 9, preventing us from analysing changes in in‐person social contacts, which likely decreased due to disease control measures. Given evidence suggesting that non‐physical social contacts can mitigate the mental health impacts of lockdowns on older adults (Arpino et al. 2021), we focused on changes in remote social contacts. *Remote social contact changes* were defined as changes in weekly social contact frequency with children, family and friends between Cov1 and Wave 9 with three categories: increased, decreased and no change. These were further distinguished into changes in real‐time contacts (video/phone call) and written contacts (email, text). Supporting Information S1: Material 3‐e describes how remote social contacts were coded.

*Covariates* included *demographic variables,* including *age* at Wave 8 (categorised as 50–64‐year‐old, 65–74‐year‐old and 75‐year‐and‐older), *sex* (female vs. male), *migration status* (UK‐born vs. non‐UK‐born) and *place of residence* (rural vs. urban, sourced from the 2011 census archive). Migration status was included on the basis that ethnic minorities and migrant communities experienced disproportionate social and economic impacts during the COVID‐19 pandemic (Platt and Warwick 2020) and may be more vulnerable to loneliness (Joshi et al. 2025a, 2025b).

### 3.3 Statistical Analysis

After investigating the descriptives, we applied a three‐step latent class (LC) approach following Vermunt’s (2010) methodology. LC analysis uncovers hidden clusters by grouping subjects into mutually exclusive subgroups, maximising heterogeneity between classes and homogeneity within classes (Lanza et al. 2003). In the first step, we conducted latent class growth (LCG) analysis to identify distinct loneliness trajectories within our sample. The outcome variable was the repeated measure of loneliness nested within individuals with time as predictors. We estimated models allowing up to 6 LCs. The determination of the optimal number of classes was based on both statistical indices and substantive grounds (Vermunt 2010). We then assigned individuals to the class for which the posterior class membership probability was highest (Step 2).

In the third step, we ran multinomial regression analysis to examine associations between the identified class membership of loneliness trajectories and demographic, general risk, pandemic‐related risk and protective factors. We used the maximum likelihood bias‐adjusted Step 3 analysis function within the LatentGOLD software to correct for classification error introduced in Step 2 (Vermunt 2010). For the multinomial regression, we employed four models, progressively adding covariates: Model 1 included demographic variables, Model 2 added general risk variables, Model 3 added pandemic‐related risk variables, and Model 4 incorporated all variables from Models 1–3 and protective variables (see Supporting Information S1: Material 7 for the model indices). Analyses were performed using LatentGOLD version 6.0. Respondents with missing values in independent variables were excluded from the Step 3 analysis (Figure 2 and Supporting Information S1: Material 4). The collinearity statistics test shows that most predictors have acceptable variance inflation factor (VIF) (all are < 3), suggesting no severe multicollinearity overall (Supporting Information S1: Material 5).

## 4 Results

Table 1 presents the sample characteristics. Loneliness prevalence was 15.4% in Wave 8, 16.5% in Wave 9, 19.7% in Cov1 and 20.5% in Cov2.

**TABLE 1:** Cross‐tabulation of loneliness trajectory classes with demographic, general risk, pandemic‐related risk and protective factors.

	C1: Not lonely	C2: Pandemic loneliness	C3: Transitioned out of loneliness	C4: Enduring loneliness	Total	Chi‐square
Variable	*N* (%)	*N* (%)	*N* (%)	*N* (%)	*N* (%)	*p*‐value
Loneliness (Wave 8)	101 (2.8%)	104 (23.6%)	217 (100%)	272 (100%)	694 (15.4%)	< 0.001
Loneliness (Wave 9)	113 (3.2%)	182 (41.4%)	172 (79.3%)	272 (100%)	739 (16.5%)	< 0.001
Loneliness (Cov1)	181 (5.1%)	346 (78.6%)	87 (40.1%)	272 (100%)	886 (19.7%)	< 0.001
Loneliness (Cov2)	199 (5.6%)	405 (92%)	47 (21.7%)	272 (100%)	923 (20.5%)	< 0.001
50–64 years old	1357 (38.1%)	172 (39.1%)	87 (40.1%)	117 (43%)	1733 (38.6%)	0.491
65–74 years old	1565 (43.9%)	188 (42.7%)	86 (39.6%)	116 (42.6%)	1955 (43.5%)	
75 years and older	641 (18%)	80 (18.2%)	44 (20.3%)	39 (14.3%)	804 (17.9%)	
Male	1652 (46.4%)	129 (29.3%)	83 (38.2%)	100 (36.8%)	1964 (43.7%)	< 0.001
Female	1911 (53.6%)	311 (70.7%)	134 (61.8%)	172 (63.2%)	2528 (56.3%)	
UK‐born	3199 (89.8%)	388 (88.2%)	190 (87.6%)	243 (89.3%)	4020 (89.5%)	0.561
Non‐UK‐born	363 (10.2%)	52 (11.8%)	27 (12.4%)	29 (10.7%)	471 (10.5%)	
Rural	1073 (30.1%)	100 (22.8%)	59 (27.2%)	66 (24.3%)	1298 (28.9%)	0.003
Urban	2488 (69.9%)	339 (77.2%)	158 (72.8%)	206 (75.7%)	3191 (71.1%)	
Low SSS	968 (27.7%)	184 (42.4%)	105 (49.3%)	141 (53.2%)	1398 (31.8%)	< 0.001
Middle SSS	1487 (42.6%)	155 (35.7%)	86 (40.4%)	82 (30.9%)	1810 (41.1%)	
High SSS	1036 (29.7%)	95 (21.9%)	22 (10.3%)	42 (15.8%)	1195 (27.1%)	
Employed	1057 (29.8%)	111 (25.4%)	64 (29.8%)	68 (25.1%)	1300 (29.1%)	< 0.001
Retired	2284 (64.3%)	289 (66.1%)	128 (59.5%)	162 (59.8%)	2863 (64.0%)	
Not‐in‐labour‐force	209 (5.9%)	37 (8.5%)	23 (10.7%)	41 (15.1%)	310 (6.9%)	
Poor self‐rated general health	552 (15.5%)	120 (27.3%)	70 (32.3%)	112 (41.2%)	854 (19.0%)	< 0.001
Good self‐rated general health	3011 (84.5%)	320 (72.7%)	147 (67.7%)	160 (58.8%)	3638 (81.0%)	
CES‐D‐8 ≥ 3	295 (8.3%)	119 (27.3%)	85 (39.4%)	134 (49.4%)	633 (14.2%)	< 0.001
CES‐D‐8 < 3	3249 (91.7%)	317 (72.7%)	131 (60.6%)	137 (50.6%)	3834 (85.8%)	
COVID‐related worries	619 (17.4%)	150 (34.2%)	79 (36.4%)	154 (56.8%)	1002 (22.3%)	< 0.001
No COVID‐related worries	2942 (82.6%)	289 (65.8%)	138 (63.6%)	117 (43.2%)	3486 (77.7%)	
Disrupted daily routines	935 (26.3%)	203 (46.3%)	70 (32.4%)	133 (49.1%)	1341 (29.9%)	< 0.001
No disrupted daily routines	2626 (73.7%)	235 (53.7%)	146 (67.6%)	138 (50.9%)	3145 (70.1%)	
COVID symptoms ≥ 2	342 (9.6%)	65 (14.8%)	33 (15.2%)	63 (23.3%)	503 (11.2%)	< 0.001
No COVID symptoms ≥ 2	3210 (90.4%)	375 (85.2%)	184 (84.8%)	207 (76.7%)	3976 (88.8%)	
Self‐isolation	872 (24.5%)	140 (31.8%)	79 (36.4%)	90 (33.1%)	1181 (26.3%)	< 0.001
No self‐isolation	2691 (75.5%)	300 (68.2%)	138 (63.6%)	182 (66.9%)	3311 (73.7%)	
Pray/meditate daily	847 (24.6%)	120 (28.4%)	60 (29.3%)	80 (30.5%)	1107 (25.5%)	0.037
Not pray/meditate daily	2602 (75.4%)	303 (71.6%)	145 (70.7%)	182 (69.5%)	3232 (74.5%)	
CASP Self‐realisation subscale	Mean: 6.44	Mean: 4.69	Mean: 5.22	Mean: 3.85	Mean: 6.05	< 0.001^†^
No partner	824 (23.2%)	217 (49.3%)	105 (48.4%)	160 (58.8%)	1306 (29.1%)	< 0.001
High partner support	2470 (69.4%)	129 (29.3%)	77 (35.5%)	52 (19.1%)	2728 (60.8%)	
Low partner support	264 (7.4%)	94 (21.4%)	35 (16.1%)	60 (22.1%)	453 (10.1%)	
Increased real‐time remote contact	1417 (39.9%)	175 (39.9%)	95 (44%)	114 (42.5%)	1801 (40.2%)	0.506
Decreased real‐time remote contact	246 (6.9%)	33 (7.5%)	19 (8.8%)	23 (8.6%)	321 (7.2%)	
No change in real‐time remote contact	1891 (53.2%)	231 (52.6%)	102 (47.2%)	131 (48.9%)	2355 (52.6%)	
Increased written remote contact	1165 (33.2%)	138 (31.7%)	77 (37%)	105 (39.9%)	1485 (33.6%)	0.178
Decreased written remote contact	275 (7.8%)	29 (6.7%)	12 (5.8%)	21 (8%)	337 (7.6%)	
No change in written remote contact	2074 (59%)	268 (61.6%)	119 (57.2%)	137 (52.1%)	2598 (58.8%)	

*Note:* ^† A^ Kruskal–Wallis test was conducted to assess differences in Self‐realisation scores among the four classes, as the distribution of the Self‐realisation score is non‐nominal.

### 4.1 Loneliness Trajectories

The four‐class solution provides the best trade‐off between model fit using statistical indices and interpretability. Supporting Information S1: Material 6‐a provides details of model fit indices and the rationale for choosing the four‐class solution. The visualisation of raw scores across four waves on the three‐item UCLA scale confirms the underlying shape of the four classes (Supporting Information S1: Material 6‐b). Most older adults (73.5%) belong to ‘not lonely’, representing individuals who self‐reported being not lonely both before and during the pandemic. We call the second largest class (12.7%) ‘pandemic loneliness’, including individuals who reported being not lonely pre‐pandemic but lonely during the pandemic. In contrast, ‘transitioned out of loneliness’ (6.9%) reported being lonely pre‐pandemic but transitioned to a nonlonely state during the pandemic. Finally, ‘enduring loneliness’ (6.8%) is characterised by loneliness both before and during the pandemic.

### 4.2 Membership in the Loneliness Trajectory Classes

**FIGURE 3:**
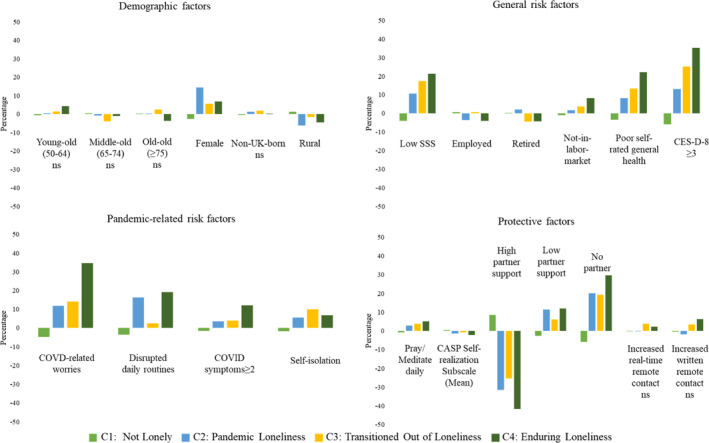
Descriptive analysis of four class memberships. *Note:* The bars represent the percentage difference of the factors within the specific class compared to the overall percentage. A positive bar indicates that older adults with that factor were over‐represented in that specific class, whereas a negative bar indicates under‐representation. Statistical significance was assessed using the chi‐square test, except for the CASP Self‐realisation subscale, for which the Kruskal–Wallis test was applied. Unless otherwise noted as ns (not significant), all factors shown are statistically significant. CES‐D‐8 ≥ 3 represents having depressive symptoms. CASP Self‐realisation subscale is for mean difference. SSS: subjective social status. CASP: Control, Autonomy, Self‐realisation and Pleasure.

Table 1 and Figure 3 present descriptive statistics on demographic, general risk, pandemic‐related risk and protective factors for the four loneliness trajectory classes. Women are significantly over‐represented in ‘pandemic loneliness’ and under‐represented in ‘not lonely’. Older adults residing in rural areas were significantly under‐represented in both ‘pandemic loneliness’ and ‘enduring loneliness’. In terms of general risk factors, those with low SSS, not‐in‐labour‐force status, reported poor self‐rated general health and depressive symptoms at baseline (Wave 8) were significantly over‐represented in ‘pandemic loneliness’, ‘transitioned out of loneliness’ and ‘enduring loneliness’. Those with pandemic‐related risk factors were significantly under‐represented in ‘not lonely’. Those reporting disrupted daily routines were notably over‐represented in both ‘pandemic loneliness’ and ‘enduring loneliness’. Lastly, those with high levels of partner support were significantly over‐represented in ‘not lonely’ and under‐represented in all three other classes.

### 4.3 Predicting Membership in Loneliness Trajectory Classes

Figure 4 (and Supporting Information S1: Material 8) presents associations between the four groups of factors and the four loneliness trajectory memberships. We compare ‘pandemic loneliness’ with ‘not lonely’ (reference group) to find out ‘who became lonely during the pandemic’ and compare ‘enduring loneliness’ with ‘not lonely’ (reference group) to identify ‘who endured loneliness’. We also compare ‘transitioned out of loneliness’ with ‘enduring loneliness’ (reference group) to answer the question of ‘who escaped loneliness’, providing an opportunity to explore potential protective factors that lifted older adults out of loneliness during the pandemic. In our analysis, ‘pandemic loneliness’ and ‘enduring loneliness’ are considered undesirable loneliness trajectories, whereas ‘not lonely’ and ‘transitioned out of loneliness’ represent desirable trajectories. We report significant factors influencing these trajectories, as well as changes in the magnitude and significance of their effects across the different models.

#### 4.3.1 Demographic Factors

The age differences in three pairs of comparisons are not statistically significant in Models 1–3, which changes in Model 4 when protective factors are added. After adjusting for other demographic factors, general risk, pandemic‐related risk and protective factors, the oldest group (75+) emerges as the least likely to belong to undesirable loneliness trajectories. For instance, compared to individuals aged 75+, those aged 50–64 are 2.32 times more likely, and those aged 65–74 are 1.78 times more likely, to belong to ‘pandemic loneliness’ (vs. ‘not lonely’); those aged 50–64 are 5.34 times more likely, and those aged 65–74 are 3.79 times more likely, to belong to ‘enduring loneliness’ (vs. ‘not lonely’); and those aged 50–64 are 75% less likely, and those aged 65–74 are 65% less likely, to belong to ‘transitioned out of loneliness’ (vs. ‘enduring loneliness’). This indicates a suppression effect, where protective factors masked the vulnerability of younger groups in the initial models and protective factors may disproportionately benefit the oldest group (75+). Sex is a significant predictor of membership in ‘pandemic loneliness’ versus ‘not lonely’. In Model 4, controlling for all other variables, women had a higher likelihood (RRR = 1.59; 95% CI: 1.11–2.29) than men of belonging to ‘pandemic loneliness’ (vs. ‘not lonely’).

FIGURE 4Forest plot showing relative risk ratios (RRRs) for predictors of class membership across four nested multinomial regression models. *Note:* Red dots indicate statistically significant RRRs at the 95% confidence level; black dots represent nonsignificant predictors. The figure is intended to illustrate the direction and changes in the strength of associations across the four models. Detailed RRRs and 95% confidence intervals for all predictors are provided in Supplementary Material 8.

**Figure:**
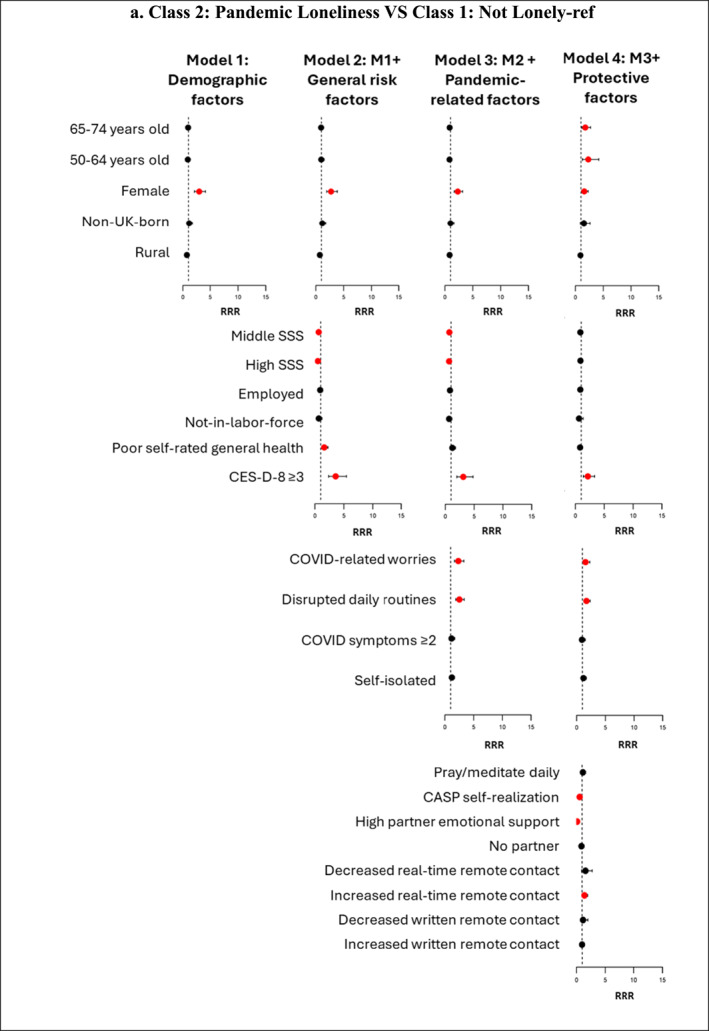

**Figure:**
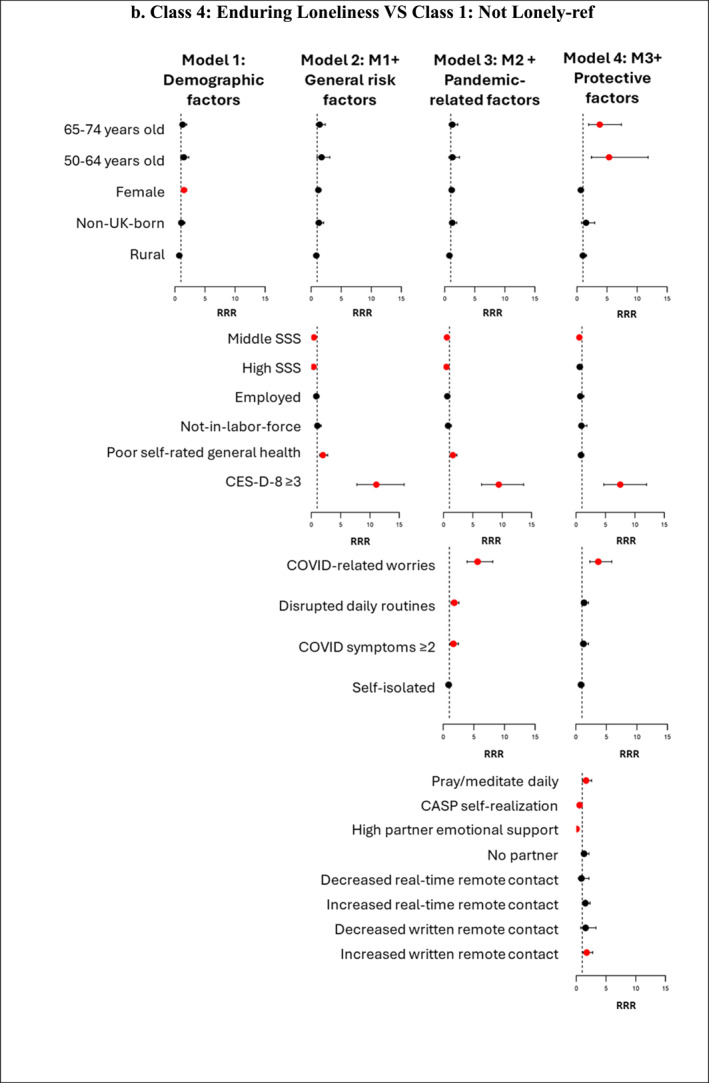

**Figure:**
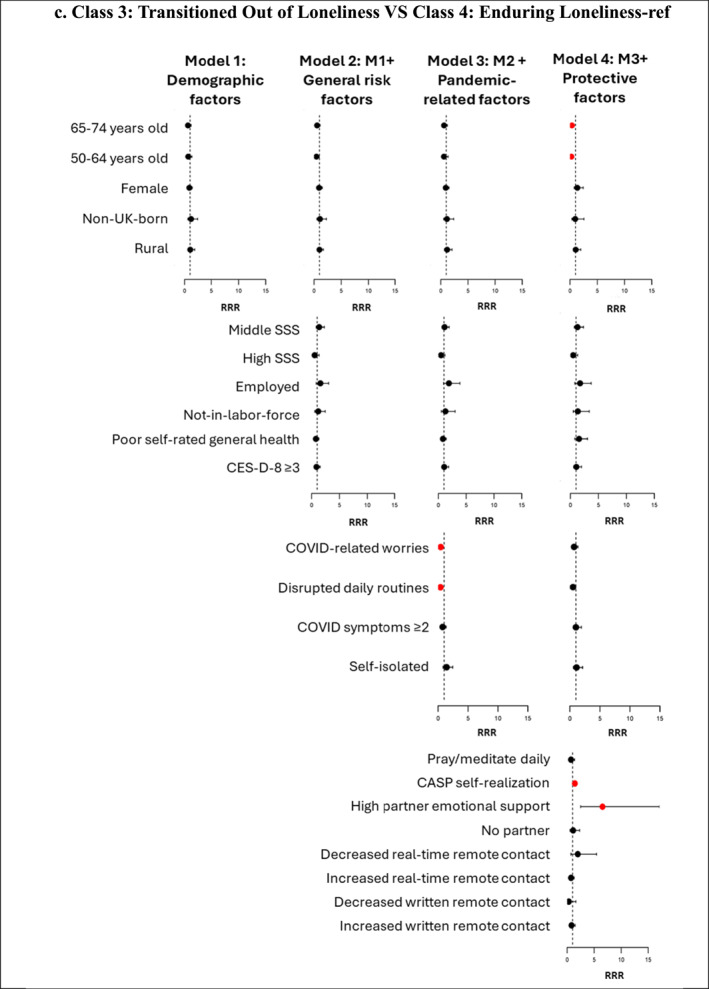

#### 4.3.2 Vulnerability Pillar: General Risk Factors

SSS at baseline (Wave 8) showed an initial protective effect against ‘pandemic loneliness’ and ‘enduring loneliness’ (vs. ‘not lonely’), but this effect diminished when protective factors were added in Model 4. For instance, in Model 3, after controlling for demographic factors, other general risk factors and pandemic‐related risk factors, individuals with high SSS, compared to low SSS, were 34% less likely to belong to ‘pandemic loneliness’ (vs. ‘not lonely’). However, this association became nonsignificant after adding protective factors in Model 4 (RRR = 0.88; 95% CI: 0.59–1.31). For membership in ‘enduring loneliness’ versus ‘not lonely’, individuals with middle SSS remained a significant protective effect even in Model 4 (RRR = 0.57; 95% CI: 0.36–0.89), though the magnitude of this effect was reduced compared to earlier models. The attenuation of the protective effect for high SSS and the partial mediation for middle SSS in Model 4 highlight the critical role of protective factors in explaining the observed associations between SSS and loneliness trajectories. SSS does not significantly predict membership in ‘transitioned out of loneliness’ versus ‘enduring loneliness’. In Model 2, where only general risk and demographic factors are considered, poor self‐rated general health at baseline was significantly associated with belonging to ‘pandemic loneliness’ and ‘enduring loneliness’ (vs. ‘not lonely’). For instance, those who reported poor health in Wave 8 were 1.61 times more likely than those who reported good health to belong to ‘pandemic loneliness’ (vs. ‘not lonely’) and 1.98 times more likely to belong to ‘enduring loneliness’ (vs. ‘not lonely’). However, these associations reversed direction and became nonsignificant in Model 4 when protective factors were added. This suggests that protective factors mediate the relationship between poor health and loneliness trajectories. Depressive symptoms (CES‐D‐8 ≥ 3) consistently show a strong and statistically significant positive association with membership in ‘pandemic loneliness’ and ‘enduring loneliness’ (vs. ‘not lonely’) across all models. In Model 4, even after adjusting for demographic, other general risk, pandemic‐related risk and protective factors, individuals experiencing depressive symptoms were 2.16 times more likely to belong to ‘pandemic loneliness’ (vs. ‘not lonely’) and 7.52 times more likely to belong to ‘enduring loneliness’ (vs. ‘not lonely’). None of the general risk factors have a significant association with ‘transitioned out of loneliness’ and ‘enduring loneliness’ memberships.

#### 4.3.3 Exposure Pillar: Pandemic‐Related Risk Factors

In Model 3, when protective factors are not accounted for, COVID‐related worries and disrupted daily routines were both significantly associated with a higher likelihood of belonging to ‘pandemic loneliness’ and ‘enduring loneliness’ (vs. ‘not lonely’) and a lower likelihood of belonging to ‘transitioned out of loneliness’ (vs. ‘enduring loneliness’). However, after adding protective factors in Model 4, notable changes in these associations emerged. For ‘pandemic loneliness’ versus ‘not lonely’, both COVID‐related worries and disrupted daily routines remained significant, but the magnitude of their effects decreased (COVID‐related worries: RRR = 2.35 in Model 3 to RRR = 1.55 in Model 4; disrupted daily routines: RRR = 2.53 in Model 3 to RRR = 1.71 in Model 4). For ‘enduring loneliness’ versus ‘not lonely’, COVID‐related worries remained a significant predictor, but disrupted daily routines became a nonsignificant predictor. For ‘transitioned out of loneliness’ versus ‘enduring loneliness’, both became nonsignificant after adding protective factors. Additionally, those with two or more COVID symptoms were 1.67 times more likely to belong to ‘enduring loneliness’ (vs. ‘not lonely’) in Model 3, which became nonsignificant in Model 4. These changes indicate that protective factors mediate the relationship between pandemic‐related stressors and loneliness trajectory outcomes.

#### 4.3.4 Capacity Pillar: Protective Factors

A higher CASP Self‐realisation score and high partner emotional support play a significant role in shielding older adults from undesirable loneliness trajectories. Each one‐point increase in the CASP Self‐realisation score is associated with a 41% lower likelihood of belonging to ‘pandemic loneliness’ (vs. ‘not lonely’), a 44% lower likelihood of belonging to ‘enduring loneliness’ (vs. ‘not lonely’) and a 42% higher likelihood of belonging to ‘transitioned out of loneliness’ (vs. ‘enduring loneliness’). Individuals with high partner emotional support, compared to those with low support, were 84% less likely to belong to ‘pandemic loneliness’ (vs. ‘not lonely’), 95% less likely to belong to ‘enduring loneliness’ (vs. ‘not lonely’) and 6.54 times more likely to belong to ‘transitioned out of loneliness’ (vs. ‘enduring loneliness’). Notably, no significant differences were observed between individuals with low partner emotional support and those with no partner across these trajectories. Regarding changes in remote contact, individuals with increased real‐time remote contact were 1.42 times more likely to belong to ‘pandemic loneliness’ (vs. ‘not lonely’), and those with increased written remote contact were 1.75 times more likely to belong to ‘enduring loneliness’ (vs. ‘not lonely’). Additionally, individuals who pray or meditate daily were 1.65 times more likely to belong to ‘enduring loneliness’ (vs. ‘not lonely’).

## 5 Discussion

We examined the heterogeneous trajectories of loneliness within the older population in England by analysing data from two pre‐pandemic waves and two peri‐pandemic waves of ELSA. There was a 3.2% increase in the prevalence of loneliness during the COVID‐19 pandemic compared to the pre‐pandemic years of 2018–2019. However, this increase was not uniform. Our analysis identified four latent classes of loneliness trajectories within older adults in England. Around 12.7% became lonely during the pandemic, whereas most older adults (73.5%) remained not lonely both before and during the pandemic. The remaining two classes were characterised by pre‐pandemic loneliness, with approximately half (6.8%) continuing to be lonely, while the other half (6.9%) transitioning to not lonely during the pandemic.

### 5.1 Age, Resilience and the Heterogeneity of Loneliness

The common assumptions that later life is inevitably marked by loneliness and that older adults are exceptionally vulnerable to loneliness during the pandemic need to be revisited. We found that in our sample, compared to their younger counterparts (aged 50–74), adults aged 75+ were less likely to become lonely during the pandemic (vs. ‘not lonely’) and less likely to experience loneliness both before and during the pandemic (vs. ‘not lonely’), whereas they were more likely to transition out of loneliness during the pandemic (vs. ‘enduring loneliness’). This corroborates with previous studies which find that in the COVID‐19 pandemic context, older age was associated with lower loneliness (Li and Wang 2020) and a higher chance of belonging to a mentally resilient class rather than an increasing burden class (Mayerl et al. 2022). According to socioemotional selectivity theory (Carstensen et al. 1999), as individuals age, they become more selective about the people with whom they interact, leading to a reduction in the size of their social network. Therefore, although older adults may have smaller social circles, the depth and emotional support within these relationships may not diminish in the absence of in‐person contacts due to COVID‐19 restrictions. Another explanation may stem from a resilience perspective. Research suggests that individuals in older age groups exhibit a remarkable capacity to adapt to impending and adverse life events. They adjust their aspirations and employ effective coping mechanisms when faced with adversity, which may enhance their ability to maintain subjective well‐being (Nicolaisen and Thorsen 2014). The age differences in loneliness trajectory memberships observed in our study became significant only after protective factors were included in the model. This suppression effect suggests that protective factors disproportionately benefit the oldest group, potentially reflecting greater resilience, more established support networks or different coping strategies (Ma and Joshi 2022).

However, notable heterogeneity exists within the older population. Certain older subgroups, such as women and those with depression before the pandemic, appeared to be particularly susceptible to unfavourable loneliness trajectories during the pandemic. Women in our sample who were not lonely before the pandemic had a heightened risk of becoming lonely during the pandemic, consistent with findings from other longitudinal studies (Ernst et al. 2022). Depression has been consistently shown to increase the risk of loneliness (Dahlberg et al. 2022), with older adults experiencing pre‐existing mental health conditions being more susceptible to the negative effects of the pandemic (Vahia et al. 2020). Our study adds to this evidence by showing that, during the pandemic, depressive symptoms were a significant risk factor for transitioning from a state of not being lonely before the pandemic to experiencing loneliness during the pandemic, as well as for remaining lonely throughout.

### 5.2 Understanding Loneliness Trajectories Through the Three‐Pillar Framework of Vulnerability, Exposure and Capacity to Respond

The existing loneliness literature consistently links low socioeconomic status and poor general health to an increased risk of loneliness (Dahlberg and McKee 2014). Our study builds on this understanding by examining the role of subjective social status (SSS) and self‐rated general health at baseline (Wave 8) in predicting loneliness trajectories during the pandemic. Initially, low SSS was significantly associated with a higher likelihood of belonging to ‘pandemic loneliness’ (vs. ‘not lonely’). However, after accounting for pandemic‐related factors, this association diminished, suggesting that SSS does not directly determine membership in ‘pandemic loneliness’ but operates through intermediary factors. SSS reflects individuals' perceptions of their position in the social hierarchy, which is often tied to both material resources (e.g., income and education) and psychosocial resources (e.g., self‐esteem and social networks) (Demakakos et al. 2008). Lower SSS may increase exposure to pandemic‐related stressors (Buffel et al. 2023), such as worries or disruptions, which directly heighten loneliness. A similar pattern emerged for self‐rated general health. Poor self‐rated health was initially significantly associated with membership in both ‘pandemic loneliness’ and ‘enduring loneliness’ (vs. ‘not lonely’). However, this relationship diminished after adjusting for pandemic‐related factors and was even reversed, though not statistically significant, after introducing protective factors. This suggests that self‐rated health does not independently predict loneliness trajectories but is mediated by situational factors, such as pandemic risks and protective resources. Mayerl et al. (2022) find that poor self‐rated health was associated with a higher chance of belonging to the ‘resilient’ class characterised by lower loneliness, depressive and anxiety symptoms, compared to more burdened classes (e.g., increasing burden class and high burden class). Our findings provide a potential explanation: Protective factors might buffer individuals with poor health from experiencing loneliness, potentially offsetting their initial vulnerability. This aligns with the assumption that poor health could elicit more attention and/or support, which could contribute to reduced loneliness during the pandemic despite existing health challenges. These findings emphasise the importance of considering both direct and indirect pathways through which SSS and self‐rated general health influence loneliness trajectories.

Disease control measures during the pandemic can have unintended consequences, triggering specific stressors and disrupting daily routines (Dahlberg 2021). Our analysis shows that worries about the future financial situation and the sufficiency of food and other essential items are associated with a higher risk of becoming lonely during the pandemic. Additionally, older people in our sample expressing such concerns were more prone to belonging to ‘enduring loneliness’ than ‘not lonely’. These worries may signal a lack of social support networks, irrespective of the pandemic, and may also indicate pre‐existing financial insecurity. Disruptions to daily routines are found to be associated with adverse outcomes, including higher levels of depression, lower quality of life and reduced life satisfaction during the pandemic (Di Gessa and Zaninotto 2023). Our findings are consistent with this evidence, showing that reductions in physical activity, changes in eating habits and sleep patterns elevate the risk of loneliness during the pandemic. We did not observe a significant association between COVID‐19 symptoms and increased loneliness, as found in a previous study (Li and Wang 2020). Therefore, it is not COVID‐19 itself—such as illness or isolation at home—that drives loneliness among older adults during the pandemic, but rather the unintended consequences, such as concerns about inadequate supplies, financial uncertainty and disrupted daily routines.

Our analysis reveals optimism about the future and high partner support as significant protective factors against undesirable loneliness trajectories during the pandemic. Positive psychosocial resources can serve as a buffer against various adverse situations, including challenges brought by the pandemic (Snyder et al. 2020). For instance, people who are optimistic generally have positive expectations for the future, which is associated with better adjustment to diverse stressors and correlated to lower feelings of loneliness (Rius‐Ottenheim et al. 2012). Our study underscores the potential importance of a strong sense of Self‐realisation, which may help prevent older adults from becoming lonely during the pandemic, protect them from enduring loneliness and facilitate their transition from pre‐pandemic loneliness to a state of not being lonely. However, as loneliness may influence individuals to view the world through a less optimistic lens, the bidirectional relationship between loneliness and optimism warrants further investigation. The protective role of partner support aligns with existing literature on loneliness, both before (Pinquart and Sörensen 2003) and during the pandemic (Bu et al. 2020). Our study reveals that higher levels of partner support prevented older adults from becoming lonely and enduring loneliness and aided in transitioning out of loneliness during the pandemic. Understandably, being able to open up and share feelings with a partner could alleviate worries and fears during uncertain times, such as a global pandemic. Furthermore, our analysis reveals that the inclusion of protective factors significantly altered the effects of pandemic‐related risk factors on loneliness trajectories. For example, the magnitude of the effect of COVID‐related worries and disrupted daily routines on ‘pandemic loneliness’ (vs. ‘not lonely’) was reduced, and their significance in predicting ‘transitioned out of loneliness’ (vs. ‘enduring loneliness’) disappeared after accounting for these protective resources. These changes underscore the buffering role of protective factors in mitigating loneliness risk during the pandemic. However, lower partner support presents a risk similar to having no partner at all, warning us that solely using marital status as an indicator of older adults' loneliness is insufficient. Simply being married does not guarantee protection from loneliness; the quality of partner support is critical (de Jong Gierveld et al. 2009). This finding reinforces the subjective nature of loneliness, which depends not merely on the presence of social relationships but on their perceived adequacy and emotional quality (C. Victor et al. 2008).

Previous research suggests that digital interactions can help alleviate loneliness (Fakoya et al. 2020), and studies conducted during the COVID‐19 pandemic indicate that non‐physical social contacts may protect older adults from increased mental health problems, such as depressive symptoms (Arpino et al. 2021). However, our findings did not support the protective role of increased remote social contact against loneliness. Instead, increased written remote contact was associated with a higher likelihood of belonging to ‘enduring loneliness’ (vs. ‘not lonely’), whereas increased real‐time remote contact was linked to a higher likelihood of belonging to ‘pandemic loneliness’ (vs. ‘not lonely’). Given the cross‐sectional nature of our multinomial regression analysis, we cannot determine the direction of these associations. It is possible that older adults who were already lonely, or who became lonely during the pandemic, were more likely to seek remote social contact, rather than remote contact contributing to increased loneliness. A similar explanation applies to the finding that daily prayer or meditation was associated with ‘enduring loneliness’ (vs. ‘not lonely’), as older adults experiencing persistent loneliness may turn to religious or spiritual practices in the absence of social support from others. Future research should employ longitudinal designs to establish causal directionality and assess whether increased remote social contact alleviates or reinforces loneliness. Additionally, examining the quality of remote social interactions is essential, as meaningful engagement may have different effects than passive communication. Further investigation into religious and spiritual coping mechanisms could help clarify whether these practices provide emotional support or indicate a lack of social connections.

### 5.3 Strengths and Limitations

This study has several strengths, including its prospective design with two time points of pre‐COVID‐19 data on loneliness, the identification of heterogeneous loneliness trajectories among the older population and the application of a theoretical framework to explore multifaceted psychosocial determinants of loneliness, including factors that may contribute to its reduction. However, there are some limitations to our study. A key limitation is the treatment of time‐varying variables as time‐fixed in our analysis. Variables measured at different time points may have been influenced by earlier conditions or events, leading to potential temporal ordering bias. Treating them as static cannot fully capture their dynamic influence on loneliness trajectories. Future studies should adopt analytical approaches that accommodate time‐varying variables and consider the temporal relationships between variables to provide a more nuanced understanding of these trajectories. Secondly, our analysis excluded individuals who were lost to follow‐up in later waves and those with missing values in outcome and predictor variables, which may have introduced a selection bias. These excluded individuals differed significantly from the included sample across several key risk factors (see Supporting Information S1: Material 4 for more details). Consequently, our final sample may over‐represent healthier, higher‐status individuals with stronger protective factors against loneliness. This selection bias could result in an underestimation of adverse loneliness outcomes and the impact of the included factors. As a result, the findings may not fully reflect the experiences of the most vulnerable older adults, limiting their generalisability to this subgroup. However, while interpreting our findings with caution and without overgeneralisation, this limitation does not diminish the study's contribution to identifying heterogeneous loneliness trajectories and their associated predictors. Thirdly, due to limitations in data availability and the need to avoid overfitting our models, our analysis did not include certain important factors that may influence loneliness, such as ethnicity, social participation, social support and more detailed migration‐related variables (e.g., length of residence). Future research should incorporate these factors to provide a more comprehensive understanding of the determinants of loneliness trajectories.

### 5.4 Implications and Conclusion

This study has implications for public health policy. Although the pandemic has undoubtedly presented significant challenges, our findings reveal the heterogeneity of these challenges in older adults' social well‐being. It is essential to refrain from stigmatising the vulnerability of all older adults and instead to acknowledge their resilience, thereby preventing unintended ageism. Pre‐existing social, economic and health inequalities need to be tackled to safeguard our older people from loneliness regardless of the presence or absence of a global pandemic. Supporting older people in maintaining their usual routines and increasing physical activity can help prevent loneliness during future pandemics or other circumstances that require isolation or lockdown measures. It is also imperative to ensure that older adults have access to basic supplies, as they were more restricted from going out during the lockdown. It is dangerous to assume that having a partner guarantees protection, as the quality of the relationship plays a crucial role and may even worsen during a pandemic. By acknowledging these multifaceted factors, policymakers and practitioners can better tailor their efforts to support older adults and reduce loneliness inequalities in the face of future crises.

## Author Contributions

**Mengxing Joshi:** conceptualisation (lead), data curation, formal analysis, funding acquisition, investigation, methodology, project administration, software, visualisation, writing – original draft, writing – review and editing. **Daniela Weber:** conceptualisation (supporting), methodology, validation, supervision, writing – review and editing. **Anne Goujon:** conceptualisation (supporting), methodology, supervision, writing – review and editing.

## Funding

This study is supported by the Economic and Social Research Council (Grant ES/P000681/1) and a Natural Environment Research Council grant. The views and opinions expressed are, however, those of the authors only and do not necessarily reflect those of the Economic and Social Research Council or the Natural Environment Research Council or the United Kingdom Research and Innovation. Neither the ESRC/NERC nor the granting authority can be held responsible for them. All authors were not precluded from accessing data in the study, and they accept responsibility to submit for publication. D.W. is a recipient of an APART‐GSK fellowship of the Austrian Academy of Sciences at the HEP division of Vienna University of Economics and Business.

## Conflicts of Interest

The authors declare no conflicts of interest.

## Supporting information


Supporting Information S1

## Data Availability

ELSA data were available through the UK Data Archive and are widely available to access in this way; therefore, our study data will not be made available for direct access.

## Endnotes

^1^
It is important to clarify our use of the term ‘older adults’ in this study. Although the conventional age threshold for categorising individuals as “older adults” is often set at 65, typically corresponding to retirement age, it is crucial to recognise that the concept of ‘old age’ is socially and culturally constructed and may vary across contexts. In this study, we employ the term ‘older adults’ in a relative and chronological sense. For instance, individuals aged 50–65 are considered relatively older compared to those under 50. This conceptualisation allows us to explore the experiences of a broader age range and to assess the impact of the COVID‐19 pandemic on social well‐being across this spectrum. From a practical standpoint, this approach also helps to maintain a sufficient sample size, particularly given the attrition observed across the four study waves.
