# Supplementary material for: Transitions in and out of Loneliness During the COVID‐19 Pandemic: A Latent Class Analysis of Older Adults in England
Source: Sociol Health Illn. 2025 Dec 21;48(1):e70138. doi: 10.1111/1467-9566.70138 (PMC12718619; doi:10.1111/1467-9566.70138)
Supplement: Supplementary file 1 — Supporting Information S1 [file SHIL-48-0-s001.docx]

Table of Contents

[Supplementary Material 1: Context of the COVID-19 pandemic in the UK 2](#_Toc213491588)

[Supplementary Material 2-a: UCLA-3 item loneliness scale dichotomization 2](#_Toc213491589)

[Supplementary Material 2-b: Latent class analysis using UCLA-3 item loneliness scale as three categories 3](#_Toc213491590)

[Supplementary Material 3: Measurements 4](#_Toc213491591)

[3-a Subjective social status (SSS) 4](#_Toc213491592)

[3-b Questions of the 8-item short version of the Center for Epidemiologic Studies Depression (CES-D) Scale 4](#_Toc213491593)

[3-c Justification for the CES-D-8 cut-point 4](#_Toc213491594)

[3-d Self-realization subscale of the Control, Autonomy, Self-realization and Pleasure (CASP-12) scale 5](#_Toc213491595)

[3-e Measurement of changes in remote social contacts 5](#_Toc213491596)

[Supplementary Material 4: Descriptive analysis of excluded respondents 7](#_Toc213491597)

[Supplementary Material 5: Multicollinearity test 8](#_Toc213491598)

[Supplementary Material 6: Latent class growth analysis 9](#_Toc213491599)

[6-a: Latent class growth analysis model selection 9](#_Toc213491600)

[6-b: Visualization of three-item UCLA loneliness raw scores showing the percentage distribution across four classes 10](#_Toc213491601)

[Supplementary Material 7: Multinomial regression analysis model indices 11](#_Toc213491602)

[Supplementary Material 8: Association between demographic, general risk, pandemic-related risk, protective factors, and membership of four loneliness trajectory classes using multinomial logistic regression models 12](#_Toc213491603)

[References: 16](#_Toc213491604)

# Supplementary Material 1: Context of the COVID-19 pandemic in the UK

The two ELSA COVID-19 substudies were conducted during two stages of the UK’s pandemic response. **The first wave (June–July 2020)** occurred shortly after the easing of the initial national lockdown (imposed in March 2020), when schools and non-essential shops had reopened and national restrictions were being gradually lifted. During this period, infection levels were relatively low (Figure S1).

**The second wave (November–December 2020)** took place during a renewed period of restrictions. A three-tier regional system had been introduced in mid-October 2020, followed by a second national lockdown from 5 November to 2 December 2020 in response to raising daily confirmed cases. This period corresponded with a sharp resurgence in COVID-19 cases. Please refer to Institute for Government (2022) for a summary timeline of UK government coronavirus lockdowns and restrictions between March 2020 and December 2021. The Figure S1 below shows the daily new confirmed COVID-19 cases per million people in the UK from February 2020 to December 2022, illustrating the broader pandemic context.

Figure S1:Daily new confirmed COVID-19 cases per million people in the UK from February 2020 to December 2022


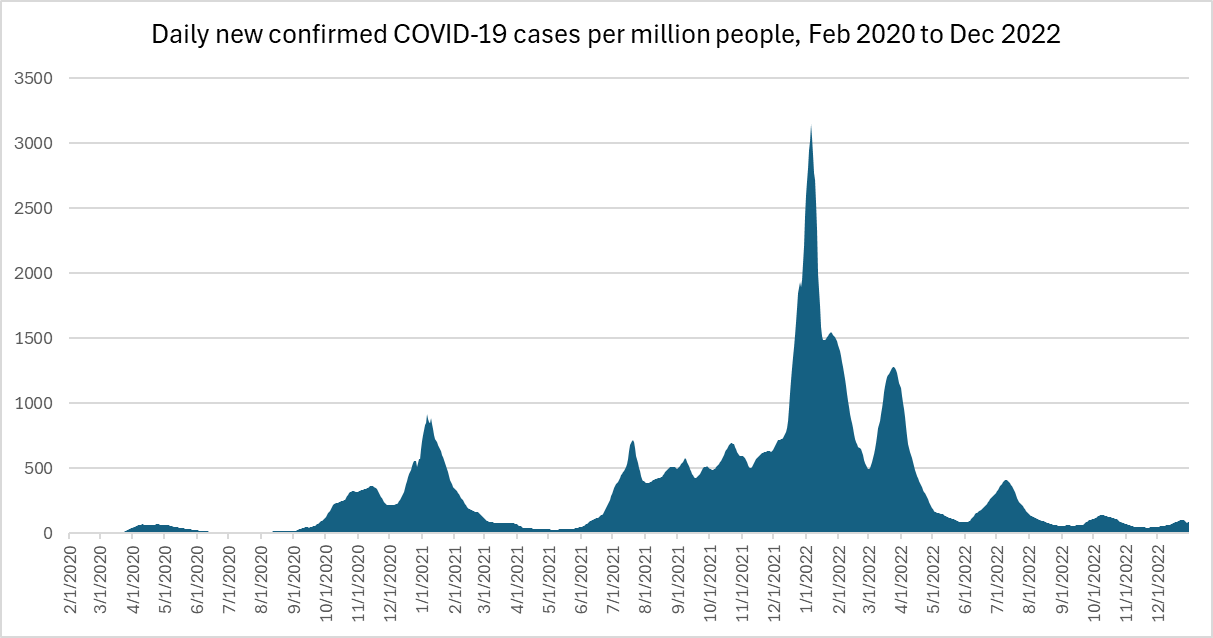


*Data source: Our World in Data* [*https://ourworldindata.org/covid-cases*](https://protect.checkpoint.com/v2/r02/___https://ourworldindata.org/covid-cases___.YzJlOmlpYXNhOmM6bzo5MWVkNjZiYTVjYjFmNjg0N2ZiNTBiM2YxZDNjZjNhYjo3OjFiNjI6ZWIxYjgyZTUxYmQ2NmJjY2YwMzg0NzhhMWNhZTg0YjNhNDJiYjgwMTNmODA1MDE2MjdjZjQ4YmE5NDc0ZjBhNjpwOkY6Tg)

# Supplementary Material 2-a: UCLA-3 item loneliness scale dichotomization

Table S1: Coding of the UCLA-3 items for dichotomization

| Category | Score | Possible answers |
| --- | --- | --- |
| Not Lonely | 3 | HHH |
|  | 4 | HHS |
|  | 5 | HHO, HSS |
| Lonely | 6 | HSO, SSS |
|  | 7 | HOO, SSO |
|  | 8 | SOO |
|  | 9 | OOO |

Note: H: Hardly ever or never. S: Some of the time. O: Often

In Waves 8 and 9, UCLA-3 item loneliness questions were included in the self-completion questionnaire, whereas in the COVID-19 Substudy, it was part of the main study survey. 90.7% of participants who completed the main interview in person in Wave 8 returning the questionnaire (Breeden et al., 2018), and 91.6% doing so in Wave 9 (Pacchiotti et al., 2021).

# Supplementary Material 2-b: Latent class analysis using UCLA-3 item loneliness scale as three categories

To explore whether a more nuanced classification would yield different insights, we also categorized loneliness into three groups: Not Lonely (scores 3–5), Moderate Loneliness (scores 6–7), and Severe Loneliness (scores 8–9). Using this classification, we conducted Latent Class Growth Analysis (LCGA) and identified four distinct loneliness trajectories (see below figure). The first class, Not Lonely (73.85%), consisted of individuals who consistently reported low loneliness levels. The second class, Not Lonely to Moderate Loneliness (13.76%), represented individuals who initially reported low loneliness but became moderately lonely over time. The third class, Moderate Loneliness to Not Lonely (9.3%), included individuals who experienced a reduction in loneliness. The fourth and smallest class, Enduring Severe Loneliness (3.1%), comprised individuals who persistently reported high loneliness.

Figure S2: Mean UCLA loneliness scores of for identified classes using the three-category classification

While the three-category approach provided some differentiation in loneliness trajectories, the Not Lonely and Not Lonely to Moderate Lonely groups were comparable to the binary classification. Given this similarity, we ultimately opted for the binary UCLA classification, as it aligns with previous research, ensuring consistency in methodological approaches. Additionally, using a binary classification enhances comparability with existing studies, facilitating potential inclusion in future systematic reviews and meta-analyses. This decision allows for greater methodological coherence, broader applicability of findings while still capturing meaningful differences in loneliness experiences.

# Supplementary Material 3: Measurements

## 3-a Subjective social status (SSS)

SSS was measured using a graphical representation of a ladder with 10 rungs, where the top of the ladder represents the best-off and the bottom denotes the worst-off. The instruction is “Think of this ladder as representing where people stand in our society. At the top of the ladder are the people who are the best off – those who have the most money, most education and best jobs. At the bottom are the people who are the worst off – who have the least money, least education, and the worst jobs or no jobs. The higher up you are on this ladder, the closer you are to the people at the very top and the lower you are, the closer you are to the people at the very bottom. Please mark a cross on the rung on the ladder where you would place yourself.” (ELSA Wave 9 Self-Completion Questionnaire, Question 32).

Previous studies show consistent and strong association between SSS and psychological functioning and health-related factors (Demakakos et al., 2018; Demakakos et al., 2008). Given that both SSS and loneliness are subjective states, a strong relationship between the two is expected.

## 3-b Questions of the 8-item short version of the Center for Epidemiologic Studies Depression (CES-D) Scale

Table S2: Wording and coding of each of the 8 items used for the CES-D scale

|  | Yes | No |
| --- | --- | --- |
| 1. Whether felt depressed much of the time during past week | 1 | 0 |
| 1. Whether felt everything they did during past week was an effort | 1 | 0 |
| 1. Whether felt their sleep was restless during past week | 1 | 0 |
| 1. Whether was happy much of the time during past week | 0 | 1 |
| 1. Whether felt lonely much of the time during past week | 1 | 0 |
| 1. Whether enjoyed life much of the time during past week | 0 | 1 |
| 1. Whether felt sad much of the time during past week | 1 | 0 |
| 1. Whether could not get going much of the time during past week | 1 | 0 |

## 3-c Justification for the CES-D-8 cut-point

At the Documentation of Affective Functioning Measures in the Health and Retirement Study, Steffick (2000) suggested a cut-point of three or more symptoms on the CES-D-8 to indicate elevated depressive symptoms. The use of this threshold has been adopted in numerous studies using ELSA data, including those examining loneliness and mental health (Pikhartova et al., 2016; Rutland-Lawes et al., 2021; Schlechter et al., 2023; Steptoe et al., 2013; White et al., 2016). Accordingly, we adopted the ≥3 cut-point in this study to maintain consistency with previous empirical research and facilitate comparability of findings across studies using ELSA and related cohorts.

A recent methodological study comparing the CES-D and the Composite International Diagnostic Interview (CIDI-SF) for assessing depression among older adults noted that there is no universally consistent threshold for defining case status on the CES-D-8 (Dang et al., 2020). Nevertheless, a score of ≥3 has been applied in the majority of studies using this instrument (Steffick, 2000). Dang et al. (2020)’s study demonstrates that, when compared with the CIDI-SF Major Depressive Episode, using a cut-point of ≥3 on the CES-D-8 yields a sensitivity of 70.2% (correctly identifying true cases) and a specificity of 84.7% (correctly identifying non-cases). This evidence supports the appropriateness of using the ≥3 threshold for identifying elevated depressive symptoms in older adult populations.

## 3-d Self-realization subscale of the Control, Autonomy, Self-realization and Pleasure (CASP-12) scale

Table S3: Wording and coding of the self-realization subscale of CASP-12 scale

|  | Never | Not often | Sometimes | Often |
| --- | --- | --- | --- | --- |
| 1. I feel full of energy these days. | 0 | 1 | 2 | 3 |
| 1. I feel life is full of opportunities. | 0 | 1 | 2 | 3 |
| 1. I feel the future looks good for me. | 0 | 1 | 2 | 3 |

## 3-e Measurement of changes in remote social contacts

To measure changes in remote social contacts, we combined relevant questions from Cov1 and Wave 9 as the questions are not exactly same and with different response options in two waves (see Figures S2 and S3 below). Remote social contact was categorized into three levels: “no weekly contact,” “once or twice a week,” and “more than three times a week.”

In Wave 9, real-time remote social contact was defined as phone communication with at least one of the following groups: children, other family members, or friends. For example, if a participant reported having phone contact with their friends more than three times a week, they were categorized as having “more than three times a week” real-time remote contact in Wave 9.

In Cov1, real-time remote contact was measured as phone OR video calls with family members OR friends/relatives. For instance, if the same participant in Cov1 reported no weekly contact via phone or video calls with family or friends/relatives, they were categorized as having “no weekly contact” in Cov1.

To calculate changes in remote social contact, we compared the frequency of real-time remote contact between the two waves. If a participant had “more than three times a week” contact in Wave 9 but “no weekly contact” in Cov1, they were classified as having decreased real-time remote contact. Conversely, if they had “more than three times a week” in Cov1 but “no weekly contact” in Wave 9, they were categorized as having increased real-time remote contact.

The same approach was applied to written remote social contact referring to communication via text message or writing/emails.

Figure S3: Visualization of changes in remote social contacts used in Wave 9


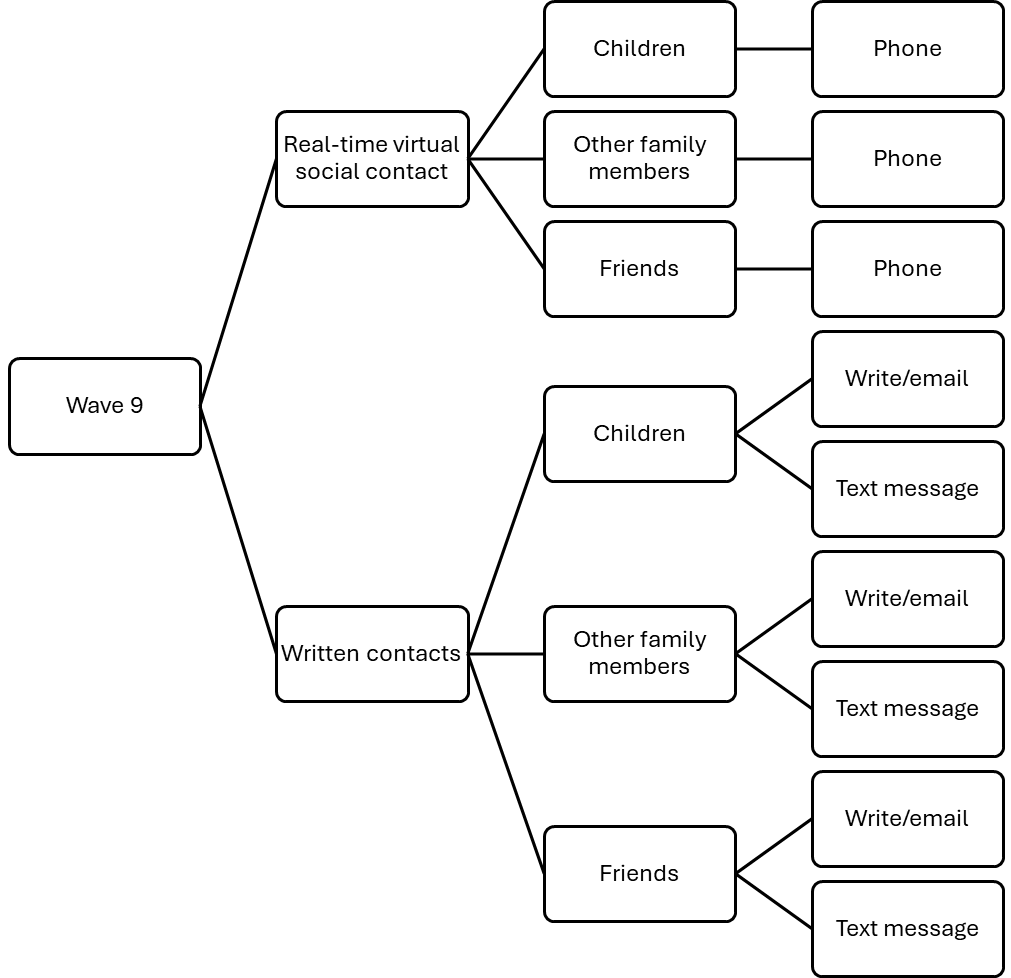


Response options: three or more times a week; once or twice a week; once or twice a month; every few months; once or twice a year; less than once a year or never.

Figure S4: Visualization of changes in remote social contacts used in Covid wave 1 (Cov 1)


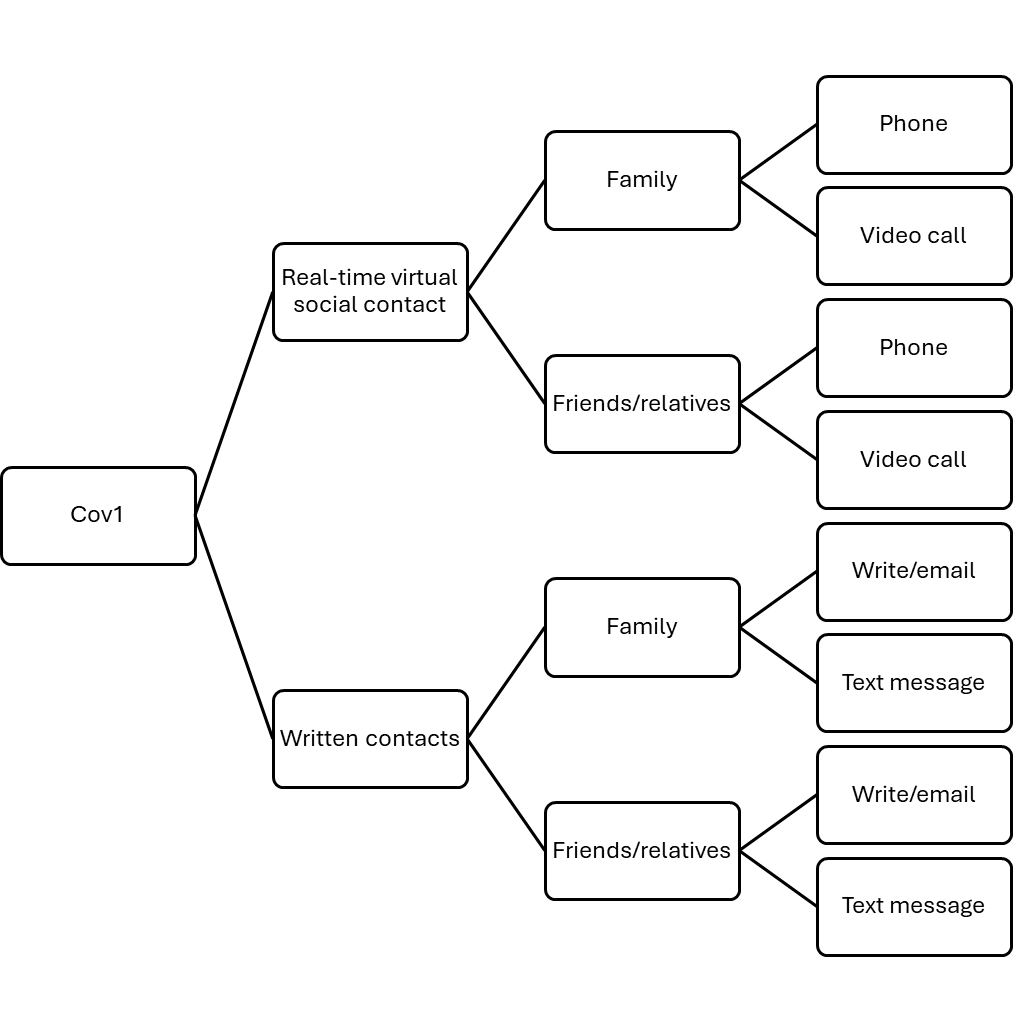


Response options: Daily, 3 to 6 times a week; once or twice a week; less than once a week or never.

# Supplementary Material 4: Descriptive analysis of excluded respondents

Table S4 Descriptive comparison on the excluded sample for Latent class analysis (LCA) and the sample included for LCA.

|  | **Excluded for LCA** | **Included for LCA** | **Chi-Square test/T-test** |
| --- | --- | --- | --- |
| Mean age | 69.91 (Average) | 67.14 | ** |
| Male | 45.2% | 43.7% | ns |
| Female | 54.8% | 56.3% |  |
| UK | 86.6% | 89.5% | ** |
| Non-UK | 13.4% | 10.5% | ** |
| Partnered | 60.1% | 71.4% | ** |
| No partner | 39.9% | 28.6% | ** |
| Average subjective social status | 57.69 | 62.68 | ** |
| Retired | 65.2% | 63.2% | ** |
| Employed | 23.5% | 28.9% |  |
| Not-in-labor | 10.7% | 6.9% |  |
| Others | 0.7% | 0.9% |  |
| Poor self-rated general health | 36.9% | 19% | ** |
| CESD-D: yes | 26.1% | 14.2% | ** |
| Loneliness at Wave 8 | 24.0% | 15.4% | ** |
| Respondents (N) | 3953 | 4492 |  |

Table S5: Descriptive comparison on the sample excluded for multinominal regression analysis due to missing independent variable data and the included sample.

|  | **Excluded** | **Included** | **Chi-Square test/T-test** |
| --- | --- | --- | --- |
| Class 1: Not lonely | 79.7% | 74.9% | ns |
| Class 2: Pandemic loneliness | 9.7% | 10.8% |  |
| Class 3: Became not lonely | 4.7% | 6.5% |  |
| Class 4: Enduring loneliness | 5.9% | 7.8% |  |
| 50-64 years old | 29.6% | 39.4% | ** |
| 65-74 years old | 39.6% | 43.9% |  |
| 75 years and older | 30.7% | 16.7% |  |
| Male | 36.9% | 44.3% | ** |
| Female | 63.1% | 55.7% |  |
| UK | 88.6% | 89.6% | ns |
| Non-UK | 11.4% | 10.4% |  |
| Urban | 74.7% | 70.8% | ns |
| Low SSS | 42.6% | 31.0% | ** |
| Middle SSS | 39.4% | 41.2% |  |
| High SSS | 18.1% | 27.8% |  |
| Retired | 66.5% | 63.8% | * |
| Employed | 24.1% | 29.5% |  |
| Not-in-labor | 9.4% | 6.7% |  |
| Poor self-rated general health | 28.3% | 18.2% | ** |
| CESD-D: yes | 20.2% | 13.7% | ** |
| COVID-related worries | 26.2% | 22.0% | ns |
| Disrupted daily routines | 22.7% | 23.0% | ns |
| COVID symptoms ≥2 | 10.1% | 11.3% | ns |
| Self-isolation | 34.0% | 25.6% | ** |
| Pray/Meditate daily | 28.4% | 25.4% | ns |
| Mean CASP Self-realization Subscale score | 5.79 | 6.07 | * |
| No partner | 33.6% | 28.7% | * |
| High partner support | 53.8% | 61.4% |  |
| Low partner support | 12.6% | 9.9% |  |
| Increased real-time remote contact | 32.6% | 40.9% | * |
| Decreased real-time remote contact | 7.0% | 7.2% |  |
| No change in real-time remote contact | 60.4% | 51.9% |  |
| Increased written remote contact | 29.8% | 33.9% | ns |
| Decreased written remote contact | 8.0% | 7.6% |  |
| No change in written contact | 62.2% | 58.5% |  |
| Respondents (N) | 371 | 4121 |  |

# Supplementary Material 5: Multicollinearity test

Table S6: Results from the multicollinearity test conducted on the independent variables used in our multinominal regression analysis.

|  | Collinearity Statistics | |
| --- | --- | --- |
|  | Tolerance | VIF |
| (Constant) |  |  |
| 65-74 years old | 0.454 | 2.204 |
| 50-64 years old | 0.331 | 3.025 |
| Female | 0.887 | 1.128 |
| Non-UK-born | 0.973 | 1.028 |
| Rural | 0.975 | 1.026 |
| Middle SSS | 0.681 | 1.469 |
| High SSS | 0.651 | 1.536 |
| Employed | 0.639 | 1.566 |
| Not-in-labor-force | 0.847 | 1.181 |
| Poor self-rated general health | 0.795 | 1.258 |
| CES-D-8 ≥3 | 0.82 | 1.22 |
| COVID-related worries | 0.822 | 1.217 |
| Disrupted daily routines | 0.888 | 1.126 |
| COVID symptoms ≥2 | 0.943 | 1.061 |
| Self-isolated | 0.877 | 1.141 |
| Pray/meditate daily | 0.948 | 1.054 |
| CASP self-realization | 0.684 | 1.463 |
| High partner emotional support | 0.333 | 3.006 |
| No partner | 0.348 | 2.876 |
| Decreased real-time remote contact | 0.918 | 1.09 |
| Increased real-time remote contact | 0.886 | 1.129 |
| Decreased written remote contact | 0.923 | 1.084 |
| Increased written remote contact | 0.894 | 1.118 |

*Note: Tolerance: The proportion of variance in a predictor that is not explained by other predictors. Low Tolerance (<0.1) indicates potential multicollinearity. Variance Inflation Factor (VIF): Quantifies how much the variance of a predictor’s coefficient is inflated due to collinearity. Thresholds: VIF < 5: Acceptable; VIF between 5–10: Moderate multicollinearity; VIF > 10: Severe multicollinearity.*

# Supplementary Material 6: Latent class growth analysis

## 6-a: Latent class growth analysis model selection

The model fit indices, as shown in the following table, reveals that the 4-class model has the lowest Bayesian Information Criterion (BIC). Vuong-Lo-Mendell-Rubin Likelihood Ratio Test (VLMART) also supports the 4-class model, indicating a significant improvement in fit over the 3-class model. While the 5-class model has the lowest AIC, its smallest class proportion is only 2%. Its limited class size may compromise the model’s practical utility. Visualizing the 4-class latent trajectories, based on the estimated means of loneliness score, clearly demonstrates distinctive patters among the four latent classes. An Entropy R^2^ value of 0.67 suggests that the 4-class model offers a moderately good classification of individuals into latent classes based on their loneliness responses. After this comprehensive assessment, we opted for the 4-class model as it strikes the best balance between model fit and practical interpretability for the study.

Table S7: Model selection criteria for the six latent class growth models

| Class | Log-likelihood | BIC(LL) | AIC(LL) | VLMRT (p-value) | Entropy R² | Smallest class proportion |
| --- | --- | --- | --- | --- | --- | --- |
| 1 | -8455·53 | 16927·88 | 16915·06 | <0·001 | 1·00 | 1·00 |
| 2 | -6798·42 | 13638·89 | 13606·84 | <0·001 | 0·80 | 0·22 |
| 3 | -6741·05 | 13549·38 | 13498·10 | <0·001 | 0·64 | 0·08 |
| **4** | **-6703·19** | **13498·89** | **13428·38** | **<0·001** | **0·67** | **0·07** |
| 5 | -6695·65 | 13509·04 | 13419·30 | 0·0005 | 0·64 | 0·02 |
| 6 | -6695·59 | 13534·16 | 13425·18 | 0·0206 | 0·41 | 0·02 |

*Notes· BIC=Bayesian Information Criterion; AIC=Akaike Information Criterion; VLMRT=Vuong-Lo-Mendell-Rubin Likelihood Ratio Test· VLMART compare an n class model with an n-1 class model·*

## 6-b: Visualization of three-item UCLA loneliness raw scores showing the percentage distribution across four classes

Figure S5: Visualization of the three-item UCLA loneliness raw scores showing the percentage distribution across four classes.


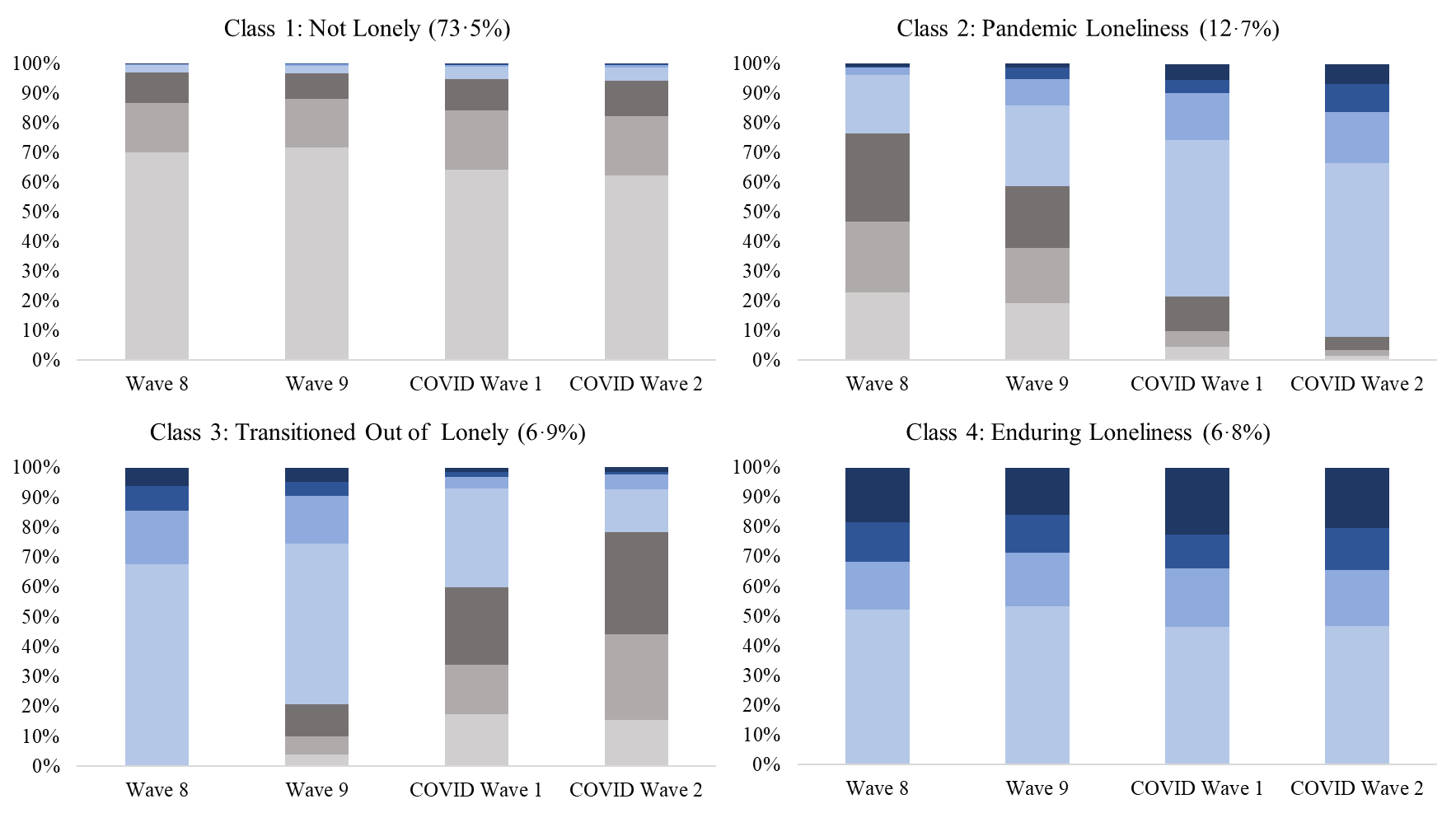


*Note*: The color g*rey indicates UCLA loneliness scores ranging from three to five from light to dark grey, categorized as not lonely. Blue represents scores ranging from six to nine from light to dark blue, classified as lonely.*

# Supplementary Material 7: Multinomial regression analysis model indices

Table S8: Multinominal regression analysis indices for the four different models investigated

|  | LL | BIC(LL) | AIC(LL) | AIC3(LL) | Class.Err. | Entropy R² |
| --- | --- | --- | --- | --- | --- | --- |
| M1: Demographic factors | -3807.3708 | 7766.1066 | 7650.7417 | 7668.7417 | 0.2649 | 0.0204 |
| M2: M1+general risk | -3450.5781 | 7202.8114 | 6973.1562 | 7009.1562 | 0.2580 | 0.1254 |
| M3: M2+pandemic-related risk factors | -3343.8425 | 7089.6599 | 6783.6850 | 6831.6850 | 0.2492 | 0.1655 |
| M4: M3+protective factors | -2893.2622 | 6385.8417 | 5930.5244 | 6002.5244 | 0.2248 | 0.2953 |

The Entropy R² values across the models provide insight into the classification accuracy and explanatory power of the predictors for loneliness trajectory outcomes. In Model 1, which includes only demographic factors, the Entropy R² is 0.0204, indicating very low classification accuracy with these predictors alone. Adding general risk factors in Model 2 substantially improves classification accuracy, increasing the Entropy R² to 0.1254. The inclusion of pandemic-related risk factors in Model 3 further enhances the model’s ability to classify outcomes, raising the Entropy R² to 0.1655. Finally, the addition of protective factors in Model 4 results in the most significant improvement, with the Entropy R² reaching 0.2953.

# Supplementary Material 8: Association between demographic, general risk, pandemic-related risk, protective factors, and membership of four loneliness trajectory classes using multinomial logistic regression models

Table S9: Output from the multinominal logistic regression analysis investigating the association between demographic, general risk, pandemic-related risk, protective factors, and membership of four loneliness trajectory classes.

|  | **Class 2: Pandemic Loneliness VS Class 1: Not Lonely-ref** | | | | | | | | | | | | | | | | | | | | | | | | | | | | | | | | |
| --- | --- | --- | --- | --- | --- | --- | --- | --- | --- | --- | --- | --- | --- | --- | --- | --- | --- | --- | --- | --- | --- | --- | --- | --- | --- | --- | --- | --- | --- | --- | --- | --- | --- |
|  | **Model: Demographic factors** | | | | | | | | **Model 2: M1+ General risk factors** | | | | | | | | | **Model 3: M2 + Pandemic-related factors** | | | | | | | | | **Model 4: M3+ Protective factors** | | | | | | |
|  | **RRR** | | **95%CI** | | | | | | **RRR** | | | **95%CI** | | | | | | **RRR** | | | **95%CI** | | | | | | **RRR** | | | **95%CI** | | | |
| 75 years and older-ref |  | |  | | |  | | |  | | |  | | |  | | |  | | |  | | |  | | |  | | |  | | |  |
| 65-74 years old | 0.95 | | 0.67 | | | 1.33 | | | 0.96 | | | 0.66 | | | 1.38 | | | 0.85 | | | 0.59 | | | 1.22 | | | 1.78 | | | 1.15 | | | 2.75 |
| 50-64 years old | 0.89 | | 0.61 | | | 1.30 | | | 0.98 | | | 0.63 | | | 1.52 | | | 0.82 | | | 0.52 | | | 1.28 | | | 2.32 | | | 1.27 | | | 4.23 |
| Female | 2.94 | | 2.09 | | | 4.15 | | | 2.74 | | | 1.94 | | | 3.85 | | | 2.31 | | | 1.67 | | | 3.19 | | | 1.59 | | | 1.11 | | | 2.29 |
| Non-UK-born | 1.13 | | 0.75 | | | 1.72 | | | 1.17 | | | 0.76 | | | 1.79 | | | 1.00 | | | 0.61 | | | 1.63 | | | 1.53 | | | 0.89 | | | 2.64 |
| Rural | 0.72 | | 0.52 | | | 1.01 | | | 0.72 | | | 0.52 | | | 0.99 | | | 0.84 | | | 0.61 | | | 1.16 | | | 0.91 | | | 0.63 | | | 1.31 |
| Low SSS-ref |  | |  | | |  | | |  | | |  | | |  | | |  | | |  | | |  | | |  | | |  | | |  |
| Middle SSS |  | |  | | |  | | | 0.66 | | | 0.49 | | | 0.90 | | | 0.71 | | | 0.51 | | | 0.98 | | | 0.86 | | | 0.61 | | | 1.23 |
| High SSS |  | |  | | |  | | | 0.51 | | | 0.35 | | | 0.76 | | | 0.66 | | | 0.45 | | | 0.96 | | | 0.88 | | | 0.59 | | | 1.31 |
| Retired-ref |  | |  | | |  | | |  | | |  | | |  | | |  | | |  | | |  | | |  | | |  | | |  |
| Employed |  | |  | | |  | | | 0.91 | | | 0.64 | | | 1.30 | | | 0.82 | | | 0.56 | | | 1.19 | | | 0.86 | | | 0.57 | | | 1.28 |
| Not-in-labor-force |  | |  | | |  | | | 0.68 | | | 0.38 | | | 1.21 | | | 0.66 | | | 0.37 | | | 1.17 | | | 0.64 | | | 0.30 | | | 1.34 |
| Poor self-rated general health |  | |  | | |  | | | 1.61 | | | 1.15 | | | 2.25 | | | 1.23 | | | 0.84 | | | 1.79 | | | 0.82 | | | 0.53 | | | 1.25 |
| CES-D-8 ≥3 |  | |  | | |  | | | 3.59 | | | 2.36 | | | 5.44 | | | 3.12 | | | 2.02 | | | 4.83 | | | 2.16 | | | 1.40 | | | 3.33 |
| COVID-related worries |  | |  | | |  | | |  | | |  | | |  | | | 2.35 | | | 1.67 | | | 3.29 | | | 1.55 | | | 1.04 | | | 2.31 |
| Disrupted daily routines |  | |  | | |  | | |  | | |  | | |  | | | 2.53 | | | 1.89 | | | 3.37 | | | 1.71 | | | 1.24 | | | 2.36 |
| COVID symptoms ≥2 |  | |  | | |  | | |  | | |  | | |  | | | 1.16 | | | 0.77 | | | 1.74 | | | 0.92 | | | 0.57 | | | 1.49 |
| Self-isolated |  | |  | | |  | | |  | | |  | | |  | | | 1.22 | | | 0.88 | | | 1.70 | | | 1.18 | | | 0.82 | | | 1.70 |
| Pray/meditate daily |  | |  | | |  | | |  | | |  | | |  | | |  | | |  | | |  | | | 1.15 | | | 0.80 | | | 1.64 |
| CASP self-realization |  | |  | | |  | | |  | | |  | | |  | | |  | | |  | | |  | | | 0.59 | | | 0.54 | | | 0.65 |
| Low partner emotional support-ref |  | |  | | |  | | |  | | |  | | |  | | |  | | |  | | |  | | |  | | |  | | |  |
| High partner emotional support |  | |  | | |  | | |  | | |  | | |  | | |  | | |  | | |  | | | 0.16 | | | 0.10 | | | 0.24 |
| No partner |  | |  | | |  | | |  | | |  | | |  | | |  | | |  | | |  | | | 0.90 | | | 0.59 | | | 1.37 |
| No change in real-time remote contact-ref |  | |  | | |  | | |  | | |  | | |  | | |  | | |  | | |  | | |  | | |  | | |  |
| Decreased real-time remote contact |  | |  | | |  | | |  | | |  | | |  | | |  | | |  | | |  | | | 1.60 | | | 0.93 | | | 2.77 |
| Increased real-time remote contact |  | |  | | |  | | |  | | |  | | |  | | |  | | |  | | |  | | | 1.42 | | | 1.03 | | | 1.97 |
| No change in written remote contact-ref |  | |  | | |  | | |  | | |  | | |  | | |  | | |  | | |  | | |  | | |  | | |  |
| Decreased written remote contact |  | |  | | |  | | |  | | |  | | |  | | |  | | |  | | |  | | | 1.18 | | | 0.69 | | | 2.01 |
| Increased written remote contact |  | |  | | |  | | |  | | |  | | |  | | |  | | |  | | |  | | | 1.01 | | | 0.69 | | | 1.46 |
| Intercept | 0.10 | | 0.07 | | | 0.14 | | | 0.11 | | | 0.08 | | | 0.17 | | | 0.08 | | | 0.05 | | | 0.13 | | | 2.67 | | | 1.25 | | | 5.72 |
|  | **Class 4: Enduring Loneliness VS Class 1: Not Lonely-ref** | | | | | | | | | | | | | | | | | | | | | | | | | | | | | | | | |
|  | **Model 1: Demographic factors** | | | | | | | **Model 2: M1+ General risk factors** | | | | | | | | | **Model 3: M2 + Pandemic-related factors** | | | | | | | | | **Model 4: M3+ Protective factors** | | | | | | | |
|  | **RRR** | | **95%CI** | | | | | **RRR** | | | **95%CI** | | | | | | **RRR** | | | **95%CI** | | | | | | **RRR** | | | **95%CI** | | | | |
| 75 years and older-ref |  | |  | |  | | |  | | |  | |  | | | |  | | |  | |  | | | |  | | |  | | |  | |
| 65-74 years old | 1.28 | | 0.83 | | 1.96 | | | 1.41 | | | 0.84 | | 2.35 | | | | 1.26 | | | 0.74 | | 2.15 | | | | 3.79 | | | 1.95 | | | 7.39 | |
| 50-64 years old | 1.48 | | 0.96 | | 2.29 | | | 1.73 | | | 0.97 | | 3.11 | | | | 1.30 | | | 0.68 | | 2.47 | | | | 5.34 | | | 2.41 | | | 11.85 | |
| Female | 1.50 | | 1.14 | | 1.99 | | | 1.20 | | | 0.86 | | 1.68 | | | | 1.16 | | | 0.82 | | 1.64 | | | | 0.65 | | | 0.41 | | | 1.02 | |
| Non-UK-born | 1.07 | | 0.69 | | 1.65 | | | 1.29 | | | 0.80 | | 2.07 | | | | 1.25 | | | 0.78 | | 2.02 | | | | 1.51 | | | 0.77 | | | 2.97 | |
| Rural | 0.73 | | 0.53 | | 1.01 | | | 0.86 | | | 0.61 | | 1.22 | | | | 0.78 | | | 0.52 | | 1.19 | | | | 0.99 | | | 0.61 | | | 1.60 | |
| Low SSS-ref |  | |  | |  | | |  | | |  | |  | | | |  | | |  | |  | | | |  | | |  | | |  | |
| Middle SSS |  | |  | |  | | | 0.45 | | | 0.31 | | 0.65 | | | | 0.58 | | | 0.40 | | 0.84 | | | | 0.57 | | | 0.36 | | | 0.89 | |
| High SSS |  | |  | |  | | | 0.39 | | | 0.25 | | 0.61 | | | | 0.50 | | | 0.31 | | 0.80 | | | | 0.62 | | | 0.34 | | | 1.13 | |
| Retired-ref |  | |  | |  | | |  | | |  | |  | | | |  | | |  | |  | | | |  | | |  | | |  | |
| Employed |  | |  | |  | | | 0.85 | | | 0.54 | | 1.35 | | | | 0.65 | | | 0.38 | | 1.09 | | | | 0.73 | | | 0.39 | | | 1.36 | |
| Not-in-labor-force |  | |  | |  | | | 1.03 | | | 0.64 | | 1.68 | | | | 0.77 | | | 0.44 | | 1.35 | | | | 0.92 | | | 0.45 | | | 1.86 | |
| Poor self-rated general health |  | |  | |  | | | 1.98 | | | 1.41 | | 2.79 | | | | 1.60 | | | 1.11 | | 2.29 | | | | 0.83 | | | 0.51 | | | 1.35 | |
| CES-D-8 ≥3 |  | |  | |  | | | 11.09 | | | 7.77 | | 15.84 | | | | 9.42 | | | 6.50 | | 13.66 | | | | 7.52 | | | 4.72 | | | 11.98 | |
| COVID-related worries |  | |  | |  | | |  | | |  | |  | | | | 5.62 | | | 3.89 | | 8.10 | | | | 3.68 | | | 2.30 | | | 5.88 | |
| Disrupted daily routines |  | |  | |  | | |  | | |  | |  | | | | 1.81 | | | 1.26 | | 2.59 | | | | 1.35 | | | 0.88 | | | 2.08 | |
| COVID symptoms ≥2 |  | |  | |  | | |  | | |  | |  | | | | 1.67 | | | 1.12 | | 2.49 | | | | 1.25 | | | 0.76 | | | 2.07 | |
| Self-isolated |  | |  | |  | | |  | | |  | |  | | | | 0.90 | | | 0.61 | | 1.32 | | | | 0.85 | | | 0.53 | | | 1.35 | |
| Pray/meditate daily |  | |  | |  | | |  | | |  | |  | | | |  | | |  | |  | | | | 1.65 | | | 1.05 | | | 2.58 | |
| CASP self-realization |  | |  | |  | | |  | | |  | |  | | | |  | | |  | |  | | | | 0.56 | | | 0.50 | | | 0.63 | |
| Partnership_2_CV1 |  | |  | |  | | |  | | |  | |  | | | |  | | |  | |  | | | |  | | |  | | |  | |
| Low partner emotional support-ref |  | |  | |  | | |  | | |  | |  | | | |  | | |  | |  | | | |  | | |  | | |  | |
| High partner emotional support |  | |  | |  | | |  | | |  | |  | | | |  | | |  | |  | | | | 0.05 | | | 0.02 | | | 0.11 | |
| No partner |  | |  | |  | | |  | | |  | |  | | | |  | | |  | |  | | | | 1.32 | | | 0.81 | | | 2.15 | |
| No change in real-time remote contact-ref |  | |  | |  | | |  | | |  | |  | | | |  | | |  | |  | | | |  | | |  | | |  | |
| Decreased real-time remote contact |  | |  | |  | | |  | | |  | |  | | | |  | | |  | |  | | | | 0.88 | | | 0.36 | | | 2.14 | |
| Increased real-time remote contact |  | |  | |  | | |  | | |  | |  | | | |  | | |  | |  | | | | 1.53 | | | 1.00 | | | 2.34 | |
| No change in written remote contact-ref |  | |  | |  | | |  | | |  | |  | | | |  | | |  | |  | | | |  | | |  | | |  | |
| Decreased written remote contact |  | |  | |  | | |  | | |  | |  | | | |  | | |  | |  | | | | 1.57 | | | 0.74 | | | 3.30 | |
| Increased written remote contact |  | |  | |  | | |  | | |  | |  | | | |  | | |  | |  | | | | 1.75 | | | 1.11 | | | 2.76 | |
| Intercept | 0.06 | | 0.04 | | 0.09 | | | 0.05 | | | 0.03 | | 0.08 | | | | 0.03 | | | 0.01 | | 0.05 | | | | 0.62 | | | 0.21 | | | 1.86 | |
|  | **Class 3: Transitioned Out of Loneliness VS Class 4: Enduring Loneliness-ref** | | | | | | | | | | | | | | | | | | | | | | | | | | | | | | | | |
|  | **Model 1: Demographic factors** | | | | | | **Model 2: M1+ General risk factors** | | | | | | | | | **Model 3: M2 + Pandemic-related factors** | | | | | | | | | **Model 4: M3+ Protective factors** | | | | | | | | |
|  | **RRR** | **95%CI** | | | | | **RRR** | | | **95%CI** | | | | | | **RRR** | | | **95%CI** | | | | | | **RRR** | | | **95%CI** | | | | | |
| 75 years and older-ref |  |  | |  | | |  | | |  | | | |  | |  | | |  | | | |  | |  | | |  | | |  | | |
| 65-74 years old | 0.63 | 0.33 | | 1.20 | | | 0.57 | | | 0.28 | | | | 1.14 | | 0.61 | | | 0.30 | | | | 1.23 | | 0.35 | | | 0.15 | | | 0.81 | | |
| 50-64 years old | 0.68 | 0.34 | | 1.34 | | | 0.45 | | | 0.21 | | | | 0.98 | | 0.58 | | | 0.25 | | | | 1.35 | | 0.25 | | | 0.09 | | | 0.74 | | |
| Female | 0.88 | 0.55 | | 1.41 | | | 0.93 | | | 0.57 | | | | 1.53 | | 0.92 | | | 0.55 | | | | 1.54 | | 1.35 | | | 0.75 | | | 2.43 | | |
| Non-UK-born | 1.19 | 0.59 | | 2.38 | | | 1.11 | | | 0.54 | | | | 2.28 | | 1.14 | | | 0.55 | | | | 2.38 | | 0.96 | | | 0.36 | | | 2.55 | | |
| Rural | 1.05 | 0.59 | | 1.85 | | | 1.01 | | | 0.59 | | | | 1.72 | | 1.18 | | | 0.67 | | | | 2.07 | | 1.05 | | | 0.56 | | | 1.97 | | |
| Low SSS-ref |  |  | |  | | |  | | |  | | | |  | |  | | |  | | | |  | |  | | |  | | |  | | |
| Middle SSS |  |  | |  | | | 1.37 | | | 0.81 | | | | 2.31 | | 1.12 | | | 0.64 | | | | 1.95 | | 1.27 | | | 0.70 | | | 2.32 | | |
| High SSS |  |  | |  | | | 0.55 | | | 0.23 | | | | 1.35 | | 0.48 | | | 0.19 | | | | 1.20 | | 0.49 | | | 0.18 | | | 1.29 | | |
| Retired-ref |  |  | |  | | |  | | |  | | | |  | |  | | |  | | | |  | |  | | |  | | |  | | |
| Employed |  |  | |  | | | 1.59 | | | 0.82 | | | | 3.07 | | 1.90 | | | 0.93 | | | | 3.87 | | 1.73 | | | 0.80 | | | 3.71 | | |
| Not-in-labor-force |  |  | |  | | | 1.20 | | | 0.58 | | | | 2.47 | | 1.28 | | | 0.54 | | | | 3.01 | | 1.32 | | | 0.52 | | | 3.36 | | |
| Poor self-rated general health |  |  | |  | | | 0.78 | | | 0.45 | | | | 1.34 | | 0.82 | | | 0.47 | | | | 1.43 | | 1.55 | | | 0.79 | | | 3.04 | | |
| CES-D-8 ≥3 |  |  | |  | | | 0.88 | | | 0.51 | | | | 1.53 | | 1.05 | | | 0.60 | | | | 1.86 | | 1.06 | | | 0.57 | | | 1.99 | | |
| COVID-related worries |  |  | |  | | |  | | |  | | | |  | | 0.44 | | | 0.26 | | | | 0.74 | | 0.70 | | | 0.36 | | | 1.35 | | |
| Disrupted daily routines |  |  | |  | | |  | | |  | | | |  | | 0.40 | | | 0.22 | | | | 0.74 | | 0.52 | | | 0.27 | | | 0.99 | | |
| COVID symptoms ≥2 |  |  | |  | | |  | | |  | | | |  | | 0.76 | | | 0.43 | | | | 1.36 | | 1.00 | | | 0.52 | | | 1.92 | | |
| Self-isolated |  |  | |  | | |  | | |  | | | |  | | 1.42 | | | 0.83 | | | | 2.45 | | 1.11 | | | 0.58 | | | 2.12 | | |
| Pray/meditate daily |  |  | |  | | |  | | |  | | | |  | |  | | |  | | | |  | | 0.71 | | | 0.37 | | | 1.39 | | |
| CASP self-realization |  |  | |  | | |  | | |  | | | |  | |  | | |  | | | |  | | 1.42 | | | 1.21 | | | 1.68 | | |
| Low partner emotional support-ref |  |  | |  | | |  | | |  | | | |  | |  | | |  | | | |  | |  | | |  | | |  | | |
| High partner emotional support |  |  | |  | | |  | | |  | | | |  | |  | | |  | | | |  | | 6.54 | | | 2.51 | | | 17.08 | | |
| No partner |  |  | |  | | |  | | |  | | | |  | |  | | |  | | | |  | | 1.11 | | | 0.53 | | | 2.31 | | |
| No change in real-time remote contact-ref |  |  | |  | | |  | | |  | | | |  | |  | | |  | | | |  | |  | | |  | | |  | | |
| Decreased real-time remote contact |  |  | |  | | |  | | |  | | | |  | |  | | |  | | | |  | | 1.97 | | | 0.71 | | | 5.45 | | |
| Increased real-time remote contact |  |  | |  | | |  | | |  | | | |  | |  | | |  | | | |  | | 0.72 | | | 0.40 | | | 1.29 | | |
| No change in written remote contact-ref |  |  | |  | | |  | | |  | | | |  | |  | | |  | | | |  | |  | | |  | | |  | | |
| Decreased written remote contact |  |  | |  | | |  | | |  | | | |  | |  | | |  | | | |  | | 0.31 | | | 0.06 | | | 1.62 | | |
| Increased written remote contact |  |  | |  | | |  | | |  | | | |  | |  | | |  | | | |  | | 0.81 | | | 0.44 | | | 1.48 | | |
| Intercept | 1.52 | 0.85 | | 2.72 | | | 1.81 | | | 0.92 | | | | 3.55 | | 2.63 | | | 1.17 | | | | 5.92 | | 0.42 | | | 0.10 | | | 1.86 | | |

*Note: For the comparison of ‘Enduring Loneliness’ and ‘Became Not Lonely’ classes, we re-run the analyses using ‘Enduring Loneliness’ as reference group.*

# References:

Breeden, J., Hussey, D., Deepchand, K., & Norton, M. (2018). *The dynamics of ageing: The 2016/2017 English Longitudinal Study of Ageing (Wave 8) Technical Report*.

Dang, L., Dong, L., & Mezuk, B. (2020). Shades of blue and gray: A comparison of the center for epidemiologic studies depression scale and the composite international diagnostic interview for assessment of depression syndrome in later life. *The Gerontologist*, *60*(4), e242-e253.

Demakakos, P., Biddulph, J. P., de Oliveira, C., Tsakos, G., & Marmot, M. G. (2018). Subjective social status and mortality: the English Longitudinal Study of Ageing. *European journal of epidemiology, 33*, 729-739.

Demakakos, P., Nazroo, J., Breeze, E., & Marmot, M. (2008). Socioeconomic status and health: the role of subjective social status. *SOCIAL SCIENCE & MEDICINE, 67*(2), 330-340.

Institute for Government (2022). *Timeline of UK government coronavirus lockdowns and restrictions*. [https://www.instituteforgovernment.org.uk/data-visualisation/timeline-coronavirus-lockdowns](https://protect.checkpoint.com/v2/r02/___https://www.instituteforgovernment.org.uk/data-visualisation/timeline-coronavirus-lockdowns___.YzJlOmlpYXNhOmM6bzo5MWVkNjZiYTVjYjFmNjg0N2ZiNTBiM2YxZDNjZjNhYjo3OmY3OWM6YzNhYTA1NTNkN2JhNmRhOWQyZWQ3MThlMjM1ZWE5MmYxMTRiZTI4MDVjYjE1Zjc3YTZlMzNiMWFmNzRlNmZjMDpwOkY6Tg)

Pacchiotti, B., Hussey, D., & Bennett, G. (2021). *The dynamics of ageing: The 2018/2019 English Longitudinal Study of Ageing (Wave 9) Technical Report*.

Pikhartova, J., Bowling, A., & Victor, C. (2016). Is loneliness in later life a self-fulfilling prophecy? *Aging & mental health, 20*(5), 543-549. https://doi.org/10.1080/13607863.2015.1023767

Rutland-Lawes, J., Wallinheimo, A. S., & Evans, S. L. (2021). Risk factors for depression during the COVID-19 pandemic: a longitudinal study in middle-aged and older adults. *BJPsych Open*, *7*(5), e161.

Steffick (2000). Documentation of Affective Functioning Measures in the Health and Retirement Study. University of Michigan. [https://hrs.isr.umich.edu/publications/biblio/5411](https://protect.checkpoint.com/v2/r02/___https://hrs.isr.umich.edu/publications/biblio/5411___.YzJlOmlpYXNhOmM6bzo5MWVkNjZiYTVjYjFmNjg0N2ZiNTBiM2YxZDNjZjNhYjo3OmNkNjg6Y2NkNDJmODQ3MjczM2FkMTE4M2YyMTE3OWU5OWI2ZTY4Y2NhMTZhZGQ2ZmZhNTY1ZmIzZWVlNzE5ZjkzNmZlNjpwOkY6Tg)

Schlechter, P., Ford, T. J., & Neufeld, S. A. (2023). The eight-item Center for Epidemiological Studies Depression Scale in the English longitudinal study of aging: Longitudinal and gender invariance, sum score models, and external associations. *Assessment*, *30*(7), 2146-2161.

Steptoe, A., Shankar, A., Demakakos, P., & Wardle, J. (2013). Social isolation, loneliness, and all-cause mortality in older men and women. *Proceedings of the National Academy of Sciences, 110*(15), 5797-5801.

White, J., Zaninotto, P., Walters, K., Kivimäki, M., Demakakos, P., Biddulph, J., Kumari, M., De Oliveira, C., Gallacher, J., & Batty, G. D. (2016). Duration of depressive symptoms and mortality risk: the English Longitudinal Study of Ageing (ELSA). *The British Journal of Psychiatry, 208*(4), 337-342.
